# Structure and Electronic Properties of TiO_2_ Nanoclusters and Dye–Nanocluster Systems Appropriate to Model Hybrid Photovoltaic or Photocatalytic Applications

**DOI:** 10.3390/nano9030357

**Published:** 2019-03-04

**Authors:** Corneliu I. Oprea, Mihai A. Gîrțu

**Affiliations:** Department of Physics and Electronics, Ovidius University of Constanța, 900527 Constanța, Romania; cornel.oprea@univ-ovidius.ro

**Keywords:** TiO_2_ clusters, electronic structure, density of states, organic dyes, dye-sensitized solar cells, penicillin, photocatalytic degradation, density functional theory

## Abstract

We report the results of a computational study of TiO_2_ nanoclusters of various sizes as well as of complex systems with various molecules adsorbed onto the clusters to set the ground for the modeling of charge transfer processes in hybrid organic–inorganic photovoltaics or photocatalytic degradation of pollutants. Despite the large number of existing computational studies of TiO_2_ clusters and in spite of the higher computing power of the typical available hardware, allowing for calculations of larger systems, there are still studies that use cluster sizes that are too small and not appropriate to address particular problems or certain complex systems relevant in photovoltaic or photocatalytic applications. By means of density functional theory (DFT) calculations, we attempt to find acceptable minimal sizes of the Ti*_n_*O_2*n*+2_H_4_ (*n* = 14, 24, 34, 44, 54) nanoclusters in correlation with the size of the adsorbed molecule and the rigidity of the backbone of the molecule to model systems and interface processes that occur in hybrid photovoltaics and photocatalysis. We illustrate various adsorption cases with a small rigid molecule based on coumarin, a larger rigid oligomethine cyanine dye with indol groups, and the penicillin V antibiotic having a flexible backbone. We find that the use of the *n* = 14 cluster to describe adsorption leads to significant distortions of both the cluster and the molecule and to unusual tridentate binding configurations not seen for larger clusters. Moreover, the significantly weaker bonding as well as the differences in the density of states and in the optical spectra suggest that the *n* = 14 cluster is a poor choice for simulating the materials used in the practical applications envisaged here. As the *n* = 24 cluster has provided mixed results, we argue that cluster sizes larger than or equal to *n* = 34 are necessary to provide the reliability required by photovoltaic and photocatalytic applications. Furthermore, the tendency to saturate the key quantities of interest when moving from *n* = 44 to *n* = 54 suggests that the largest cluster may bring little improvement at a significantly higher computational cost.

## 1. Introduction

Nanostructured titanium dioxide is well-known for its numerous and diverse applications [1,2]. Examples of such new uses are in photocatalytic degradation of pollutants [2,3,4], in dye-sensitized solar cells, for energy conversion [5,6], in sensor electronics (for detection of gases, chemicals, or biological materials) [7], in coatings with antibacterial, anticorrosion, antifogging, or self-cleaning properties [8], in drug delivery [9], etc. Due to its abundance, nontoxicity, and high stability under a variety of conditions, titanium dioxide is likely to remain the material of choice for the near future [1].

The physical and chemical properties of TiO_2_ nanocrystals, dictated to a large extent by their electronic structure, are influenced by the size, shape, organization, and surface properties of the nanoparticles [10,11]. Consequently, understanding the structure-property correlations for TiO_2_ nanostructured materials is crucial for optimizing their use in practical applications; however, it remains a challenging task even after decades of extensive studies. Density functional theory (DFT) [12,13,14] has, over the past two decades, been the method of choice for most computational studies and quite successful at predicting TiO_2_ properties and explaining experimental data. Despite some inherent difficulties [14] and limitations [15], DFT methods have been able to treat molecular systems of relatively large sizes, of up to several hundreds of atoms, with moderate computational costs [16,17].

Computational studies have discussed the electronic properties of bulk TiO_2_ polymorphs [18], the structure and reactivity of anatase surfaces, and the modeling of bare and sensitized TiO_2_ nanoparticles [16,17]. It was found that rutile is thermodynamically the most stable bulk phase, whereas anatase is very common and stable in nanomaterials [17,19], displaying the highest photocatalytic activity [20,21]. As anatase is the most interesting phase of TiO_2_ for photovoltaic and photocatalytic applications, it will be the focus of our interest in the following.

In photovoltaic and photocatalytic applications, the key role is played by some electronic properties. For instance, the energy level alignment between the conduction or valence band edges of TiO_2_ with the ground and excited states of the adsorbed molecule or with the redox level of the electrolyte determine whether a process can take place or not. To investigate the interaction of the individual dye with the TiO_2_ surface it is natural to use the cluster approach [22], whereas a periodic approach is to be preferred for studying interfaces of periodic crystalline materials, such as perovskites, on TiO_2_.

Although the TiO_2_ nanoclusters have been extensively studied theoretically, as reported in recent reviews [16,17], there are still some different perspectives regarding the proper cluster size needed to tackle a particular problem or a certain complex system. For instance, Peng et al. [23] used only a simple TiO_6_H_5_ complex to model the anchoring of the dye to the oxide, with the belief that the simple complex would be ‘sufficient for the investigation of geometries, excitations, and electronic structures of the as-investigated dyes before and after binding’. However, they also note that, to study other TiO_2_ to dyes interactions, their model should be used with precautions [23]. Zhang et al. [24] used the same model, perpetuating the claim that it was suitable for the investigation of organic dyes bound to TiO_2_. Both studies could only consider bidentate chelate anchoring, in contrast with the experimental and computational data (reviewed in [25]), favoring the bidentate bridging binding of the carboxyl group to the oxide.

Chen et al. [26] chose a Ti_5_O_20_H_22_ model system to describe the binding of several ruthenium sensitizers to the (101) anatase surface, invoking the size limitations needed to adopt a larger basis set. Although they admitted that various models, ranging from 5 to 20 Ti atoms, had been used, showing that the contributions to absorption were not equivalent [26], they went on and used the smallest cluster. There was no discussion of the impact of using one single layer of atoms and of a large number of H atoms to saturate the covalent bonds of O atoms.

Wang et al. [27] used a (TiO_2_)_6_ cluster adopted in a previous work, which in fact proposed either a complex with only one Ti atom [24] or a cluster with five Ti atoms [26]. The work influenced Mendizabal et al. [28], who used a cluster with six Ti atoms, Ti_6_O_21_H_18_, cut from the anatase crystal and completed with H atoms to end dangling bonds, and Manzoor et al. [29], who made use of a (TiO_2_)_6_ cluster, claiming that it had been ‘extensively employed as a model semiconductor nanoparticle in several studies’. None of these three studies discussed the anchoring, although it has a major role in the charge injection from the dye into the semiconducting oxide.

Analyzing the dependence of the electronic properties of the dye–oxide system on the (TiO_2_)*_n_* nanoclusters’ size, with *n* = 1, 2, 3, 6, 9, 15, and 38, Sánchez-de-Armas et al. [30] concluded that the minimal TiO_4_H_4_ model could reproduce the red shift of the optical spectra upon binding to TiO_2_. However, they recognized that the electronic structure of the system did change for larger systems, with the orbital on the dye responsible for the excitation moving from below the conduction band edge for the minimal model to well into the conduction band for larger clusters. They concluded that the *n* = 6 model could provide only a semi-quantitative simulation of all the features in the electronic structure of the complex alizarin–TiO_2_ system [30]. The same authors argued in subsequent papers that the *n* = 9 cluster was suitable for larger coumarin-based dyes [31], whereas the *n* = 15 cluster had the minimal size to provide a complete picture in the case of the smaller catechol molecule binding to the substrate [32].

Early in-depth DFT computational studies of TiO_2_ clusters were performed by Persson et al. [33,34,35], whose strategy for *n* equal to 16 and 38 was to remove selected atoms from the (101) surfaces to keep the cluster neutral and stoichiometric. They also performed systematic simulations with the cluster size ranging from *n* = 16 to 68 [36,37] and from *n* = 32 to 122 [38,39]. Independently, Jug and co-workers [40] had a different approach, in which they saturated all peripheral oxygen atoms of the clusters with hydrogen atoms and all less than fivefold coordinated titanium atoms with OH groups. They studied (TiO_2_)*_n_*(H_2_O)*_m_* clusters, with *n* = 33–132 and *m* = 17–48 [40].

Starting from the original concept of Persson and co-workers [35,36], De Angelis et al. used a cluster with *n* = 38 [41,42,43] and with *n* = 82 [44,45]. Both clusters were shown to represent a good trade-off between accuracy and computational convenience and nicely reproduced the main electronic characteristics of TiO_2_ nanoparticles [41,43], the larger one being used to check the accuracy of the results [46,47]. Larger clusters have also been studied computationally. A study of clusters with *n* between 58 and 449 and different truncated-bipyramidal shapes chose to saturate the under-coordinated surface atoms by dissociated water molecules [48]. Later, De Angelis and co-workers examined clusters of *n* = 367 and 411 [49].

In the context of photovoltaic applications [50,51], we showed that, when dealing with small molecules, an *n* = 24 cluster provides a reasonable compromise between accuracy and computational costs [52,53,54]. In contrast, when studying the photocatalytic activity of TiO_2_ under UV and visible light [55], we examined the adsorption of various common antibiotics onto TiO_2_ nanoclusters, and found that the penicillin molecule is strongly distorted when binding to the substrate [56].

In summary, despite the large number of computational studies reported so far for TiO_2_ clusters, there are still studies that use cluster sizes that are not appropriate to address particular problems or certain complex systems. To find acceptable minimal sizes of the TiO_2_ nanoclusters, one should answer questions such as: Should the size of the cluster be chosen in correlation with the size of the adsorbed molecule? Does the rigidity of the backbone of the molecule play a role in choosing the appropriate cluster? Additional questions regard the way the anchoring of the adsorbed molecule affects the structure and the optical and charge transfer properties of the system.

In an attempt to find answers to these questions, we report here computational studies of five different Ti*_n_*O_2*n*+2_H_4_ clusters, with *n* = 14, 24, 34, 44, and 54, to better understand size effects on optimized geometries and charge transfers. Our goal is to explore the structure, the anchoring, electronic spectrum, and optical properties, and the charge injection mechanisms from the adsorbed molecule to such titanium oxide clusters. We choose as adsorbed molecules simple and well-known systems, such as the coumarine-based dye, C343 [52], and penicillin V [56], as well as new molecules, such as OMCD1 [54,57].

## 2. Materials and Methods

The titanium oxide clusters reported here, regardless of size, were initially cut from the experimental anatase structure [58] to model the (101) surface. In order to ensure the charge neutrality in the presence of under-coordinated Ti atoms [48,59,60], four hydrogen atoms were used to solve the dangling bonds of the oxygen atoms bound to the two Ti atoms at the periphery of the Ti_14_O_30_H_4_ cluster, or to the three-fold coordinated Ti atoms in the corners of Ti_24_O_50_H_4_, Ti_34_O_70_H_4_, Ti_44_O_90_H_4_, and Ti_54_O_110_H_4_ clusters. These structures, for the molecules investigated, C343, OMCD1, and penicillin V, both isolated and adsorbed on the cluster, were energy minimized by DFT [12,13,14] calculations using the hybrid B3LYP exchange-correlation functional [61,62,63] and the split valence 3-21G(d) basis set with polarization functions [64,65].

For the density of electronic states, single-point calculations were performed using the hybrid B3LYP functional with the effective core potentials (ECPs) for Ti, P, S, and I atoms and double-ζ quality basis functions for all atoms via LANL2DZ [66] and accounting for aqueous solvent effects via the conductor-like polarizable continuum model (C-PCM) [67]. The cavity used in the C-PCM calculation was built from spheres centered on heavy nuclei based on the United Atom for Hartree–Fock procedure [68]. The lowest 50 singlet-to-singlet electronic transitions were calculated by Time-Dependent DFT (TD-DFT) [69]. Calculations were performed with the GAUSSIAN09 quantum chemistry package [70], whereas the projection of the density of states and the UV–Vis spectra on different system components were obtained with GaussSum [71].

## 3. Results

The results of our computational study are reported in three subsections. The first is dedicated to the clusters alone, and the following two to the clusters with adsorbed rigid and flexible molecules, respectively. In each subsection, we start from the optimized geometry and then discuss the electronic spectrum.

### 3.1. Structure and Properties of TiO_2_ Nanoclusters

**Figure 1:**
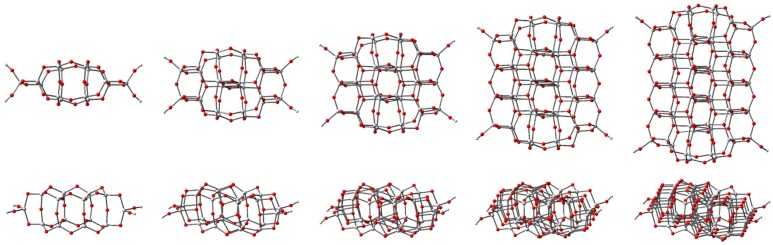
Top and lateral views of the optimized structures of the Ti_14_O_30_H_4_, Ti_24_O_50_H_4_, Ti_34_O_70_H_4_, Ti_44_O_90_H_4_, and Ti_54_O_110_H_4_ nanoclusters (from left to right, respectively) modeling the anatase titania (101) surface. The geometry optimization was performed by density functional theory (DFT) at the B3LYP/3-21G(d) level. Atom colors are grey for Ti, red for O, and light grey for H. In the top view, the widths and the lengths reported in Table 1 are measured horizontally and vertically, respectively, as shown in Figure S1 of the Supplementary Materials.

We performed geometry optimization of model clusters with a slight deviation from the TiO_2_ stoichiometry to avoid the problem of the surface states in the gap. We introduced H atoms or –OH groups to terminate the dangling bonds at the periphery, resulting in the Ti_14_O_30_H_4_, Ti_24_O_50_H_4_, Ti_34_O_70_H_4_, Ti_44_O_90_H_4_, and Ti_54_O_110_H_4_ model clusters (Ti*_n_*O_2*n*+2_H_4_). This approach resulted in compact structures with 4-, 5-, and 6-fold coordinated Ti ions, together with 2- and 3-fold coordinated oxygen atoms [48,52]. Following the geometry optimization, the structure is slightly distorted (see Figure 1) to minimize the surface stresses.

One way to examine the distortion of the lattice is by considering the average value of the Ti–O distance and its corresponding standard deviation, for the various clusters, and to compare them to the experimental values for the bulk [72], as shown in Table 1. The average distance is consistently smaller than the experimental value of 1.950 Å, valid for the bulk oxide, which has two different Ti–O distances for axial and equatorial bonds. Important to note is that the average Ti–O distance increases with the cluster size, the distortion being stronger for smaller clusters, as expected. The lattice distortion is also revealed by the distribution of the Ti–O distances, which widens significantly compared to the bulk. Moreover, the range for values of the Ti–O distances grows with cluster size. For smaller clusters, the surface-to-bulk ratio is larger, as shown below, and the distribution of distances narrower. As the cluster size increases, so does the difference between the surface and internal atoms, as the bonds are more distorted at the surface and less inside the cluster.

The values presented in Table 1 are consistent with the results of DFT calculations for stoichiometric (TiO_2_)*_n_* clusters, with *n* between 16 and 68, by other groups [36]. We also note that the values reported earlier [52,53] for *n* = 24, 34, 44 have slight deviations from those presented here due to the different DFT functionals and basis sets used.

Another way to examine the distortion of the lattice is by looking at the length and width of the various clusters and comparing those values to the corresponding distances measured in the bulk, as displayed in Table 1. The length and width of the various clusters were determined between peripheral Ti as well as O atoms.

The width of the clusters was measured as the amount the distance between the peripheral Ti atoms fluctuates from cluster to cluster with respect to the 12.04 Å value in the bulk. However, it can be observed that, despite the fluctuations, the width approaches the bulk value as the cluster size increases, as one would expect. When measuring the width between peripheral O atoms, the values are consistently larger than the one for the bulk, the range of width values being narrower.

In contrast to the width, the length of the clusters, measured between peripheral Ti atoms, is consistently smaller than the corresponding values for the bulk. In the case of the cluster lengths, the tendency is, again, to approach the values for the bulk when the cluster size increases. The lengths measured between peripheral O atoms also follow this tendency.

**Figure 2:**
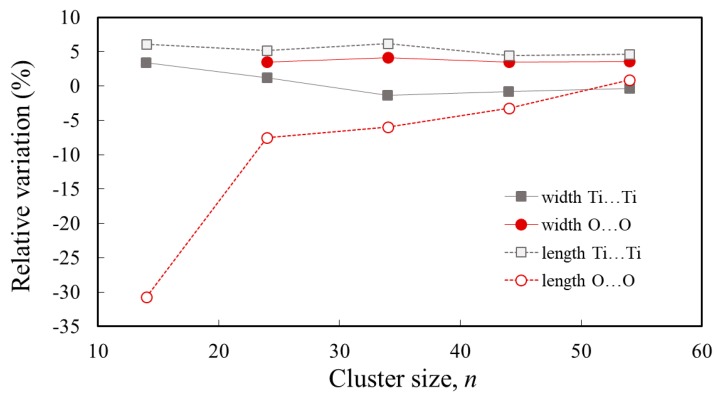
The relative cluster width, $(w_{n}-w_{\mathrm{bulk}})/w_{\mathrm{bulk}},$ and length, $(l_{n}-l_{\mathrm{bulk}})/l_{\mathrm{bulk}},$ of Ti*_n_*O_2*n*+2_H_4_, nanoclusters (where *n* = 14, 24, 34, 44, 54) with respect to the corresponding value for the bulk anatase TiO_2_ [72]. The distances are measured between peripheral Ti (grey) or O (red) atoms.

Examining in parallel the width and the length of the clusters, it can be noticed that the two quantities have opposing trends, particularly when measuring the distances between peripheral O atoms. As the cluster size increases, the width approaches the bulk value from above, whereas the length tends to the bulk from below. This tendency is illustrated in the plot of the relative variation of the cluster width and length with respect to the bulk, displayed in Figure 2.

The dependence on *n* of the relative cluster lengths shows a tendency to converge towards the bulk value, as expected. The relative variations of the cluster length (which takes values over a larger range when measuring the distance between peripheral O atoms) varies in opposition with the cluster width. The distortion allows for larger distances along one direction and shorter distances along the perpendicular direction. Overall, the distortion of the clusters is extremely large for *n* = 14, below ±10% for *n* ≥24, and less than ±5% for *n* = 54.

Earlier in this section, we stated that the distribution of Ti–O distances widens for larger *n*, when the surface-to-bulk ratio decreases. To support this statement, we present in Table 2 the numbers of Ti and O atoms of various coordination as well as the surface-to-bulk ratio, determined as the fraction of under-coordinated atoms [36]. The number of bulk (6-fold coordinated) Ti atoms increases slowly with *n*, as the clusters studied here are intended for modeling interface phenomena in hybrid photovoltaic or photocatalytic applications. For such applications, the most important anatase surface is (101) [21], already shown in Figure 1. Consequently, the clusters modeled here are extended along one direction of that particular surface and not inside, towards the bulk.

**Figure 3:**
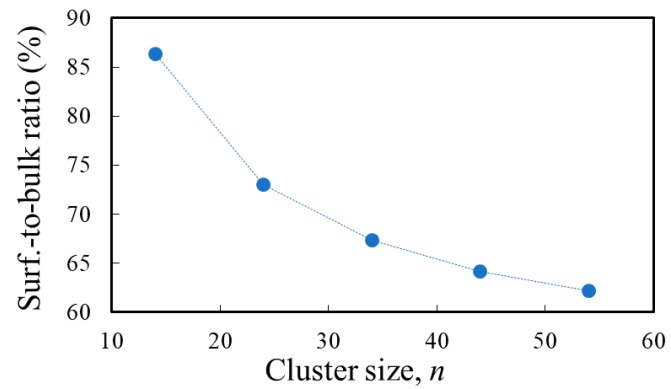
The surface-to-bulk ratio (defined as the fraction of under-coordinated atoms) dependence on the size of Ti*_n_*O_2*n*+2_H_4_ clusters (*n* = 14, 24, 34, 44, 54), geometry optimized by DFT/B3LYP/3-21G(d) in vacuum.

The surface-to-bulk ratio is also displayed in Figure 3. It can be seen that the slope of the curve decreases with *n* and there is a slight tendency of saturation for the particular type of surface cluster of interest here. The small clusters have a large number of under-coordinated atoms, which are likely to affect the electronic and optical properties of the system. Although still far from the bulk, the clusters with *n* ≥44, displaying the levelling-off of the surface-to-bulk ratio, are likely to be better candidates for describing interface phenomena.

**Figure 4:**
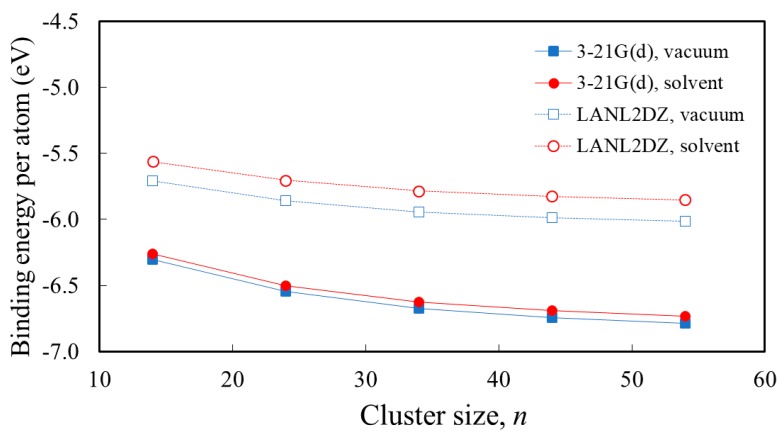
Dependence of the binding energy per atom, $\left[E_{n}-nE_{\mathrm{Ti}}-\left(2n+2\right)E_{O}-4E_{H}\right]/\left(3n+6\right)$, on the size of Ti*_n_*O_2*n*+2_H_4_ clusters (*n* = 14, 24, 34, 44, 54), calculated by B3LYP functional and 3-21G(d) or LANL2DZ basis sets in vacuum or in water solvent.

The next step from the analysis of the optimized geometry is to compare the various clusters in terms of energy. For that reason, we present in Figure 4 the binding energy per atom, determined as the difference between the energy of the Ti*_n_*O_2*n*+2_H_4_ cluster and the sum of the calculated energies of the constituents (i.e., of the *n* Ti atoms, 2*n*+2 O atoms, and four H atoms), all divided by the total number of atoms (3*n* + 6).

Regardless of the basis sets used and whether the calculation was performed in vacuum or in water solvent, the binding energy per atom increases with *n*. This result is expected, because the number of fully coordinated atoms increases with the cluster size and, therefore, the binding becomes stronger for the larger clusters. Important to notice is also the fact that the value of the binding energy per atom is levelling-off at high *n*, with less than 0.04 eV differences for *n* ≥ 44.

For each cluster, we report in Table 3 the edge of the valence band, considered at the energy of the highest occupied molecular orbital (HOMO), and the edge of the conduction band, at the lowest unoccupied molecular orbital (LUMO). The geometry relaxation leads to band gaps of 4.38–5.15 eV, values that are sensitive to the DFT functional and basis set used. For that reason, the band gaps presented here have slight deviations from those reported earlier for *n* = 24, 34, 44 [52,53].

Overall, the values obtained for the band gap are larger than the experimental value of ~3.2 eV for anatase titania [50,73]. The TiO_2_ band gap has been overestimated by many calculations of model clusters, particularly when using hybrid DFT functionals [17,74]. For instance, for the (TiO_2_)_38_ cluster, values of 3.5 eV were obtained from configuration interaction computations using about 3000 configurations [33], whereas a band gap of 3.78 eV was determined using DFT methods [41]. It has to be noticed, however, that the configuration interaction (CI) calculation was for a structure cut from the bulk, with no geometry optimization [33], and that the DFT calculation was performed on a structure optimized by Car–Parrinello molecular dynamics [41], and not by DFT, as in our calculation. Also, the two missing O atoms in the *n* = 38 model are likely to affect the valence band states, as we shall demonstrate later in this section, possibly leading to a smaller calculated gap.

The values presented in Table 3 are, overall, consistent with the results of DFT calculations for stoichiometric (TiO_2_)*_n_* clusters, with *n* between 16 and 60, for which the band gap was determined to be between 4.55 and 4.94 eV, with an anomalous value of 4.81 eV for *n* = 68 [36]. We note that the values reported by us earlier [52,53] for *n* = 24, 34, 44 have slight deviations from those presented here, due to the different DFT functionals and basis sets used.

**Figure 5:**
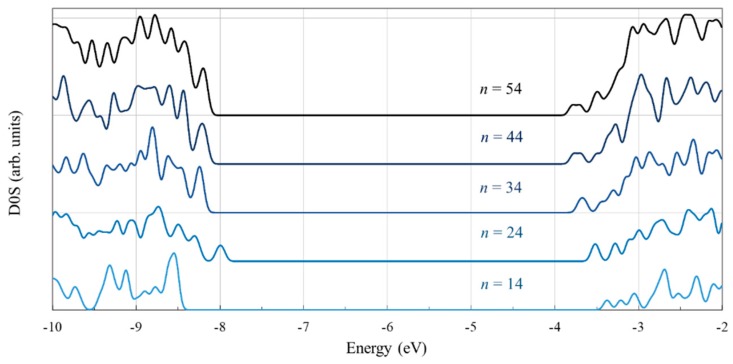
The density of states (in arbitrary units) of the Ti*_n_*O_2*n*+2_H_4_ clusters (*n* = 14, 24, 34, 44, 54), calculated at the DFT/B3LYP/LANL2DZ level in water solvent. Energy levels were convoluted with Gaussian distributions with a full width at half maximum (FWHM) of 0.1 eV.

Our calculations show that the band gap is smallest, closest to the experimental value, for the largest cluster. The gap does not vary monotonically, likely because of the particular symmetry of the *n* = 24 cluster. The density of states displayed in Figure 5 reflects the decrease in the band gap with increasing cluster size and reveals the anomaly for *n* = 24. It can be seen that the peak located at about −8 eV, which consists of the top two states in the valence band (of −7.97 and −8.02 eV, to be precise), is relatively distanced from the adjacent lower energy states, situated at −8.25 eV. This anomaly comes from the stronger electron localization on the two central oxygen atoms, which raises the energy of the orbitals by about 0.25 eV away from the next lower states. For the other clusters, the extra localization does not take place and the four highest states in the valence band are all grouped within a narrow 0.035 eV energy interval.

**Figure 6:**
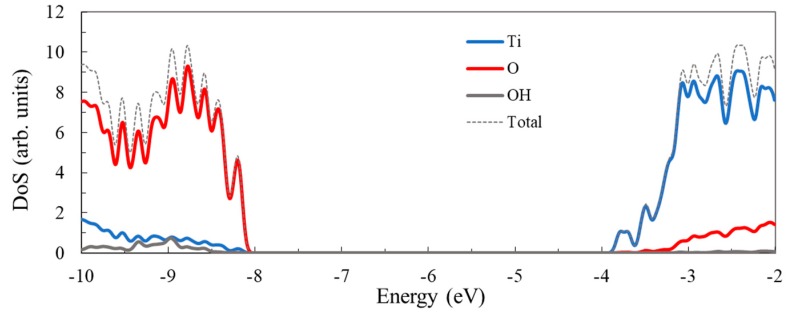
Total density of states (DoS) of the Ti_54_O_110_H_4_ cluster, calculated at the DFT/B3LYP/LANL2DZ level in water, showing, separately, the contribution of Ti atoms, O atoms, and –OH groups. Energy levels were convoluted with Gaussian distributions with an FWHM of 0.1 eV.

To better understand the nature of the states in the valence and conduction band we examine more closely the density of the state for the cluster with *n* = 54, as displayed in Figure 6. The valence band is dominated by the contribution of the O atoms. In contrast, the key contribution in the conduction band comes from the Ti atoms, the participation of the oxygen atoms being small. Worth noting is that the introduction of the hydrogen atoms, which end four dangling bonds, has a minor contribution to the overall density of states but plays an important role in removing from the gap the states localized at the surface.

The density of states curves indicate that the relative weight of the O and Ti atoms in the key molecular orbitals is consistent with the contribution of those atoms for the entire corresponding bands. Specifically, the peak at the valence band edge (the HOMO located just below −8 eV) shows a strong O contribution, whereas the peak at the conduction band edge (the LUMO situated above −4 eV) has a significant Ti weight.

**Figure 7:**
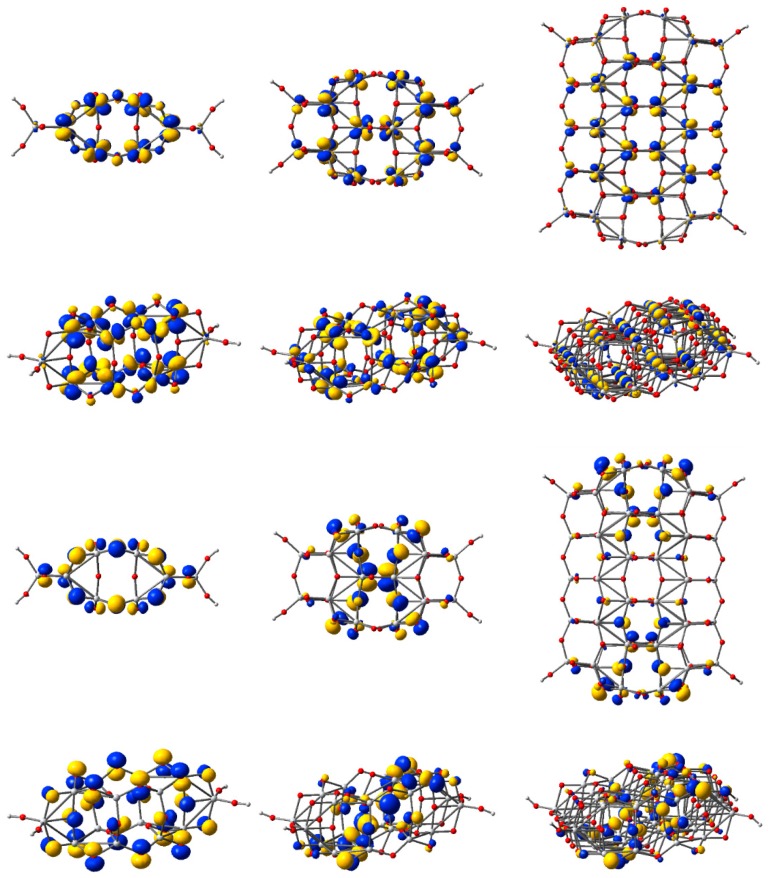
Isodensity surfaces (0.03 e/bohr^3^) of the key molecular orbitals of Ti*_n_*O_2*n*+2_H_4_ clusters (*n* = 14, 24, 54, from left to right, respectively), calculated by DFT at the B3LYP/LANL2DZ level in water solvent. The first two rows display the top and a perspective view of the LUMOs, whereas the bottom two rows show the top and a perspective view of the HOMOs. Atom colors: Ti, grey; O, red; and H, light grey.

To visually illustrate the nature of the key orbitals discussed above, we display in Figure 7 the electronic density of the highest occupied molecular orbitals and the lowest unoccupied molecular orbitals for three of the five clusters. The two orbitals are especially important as they describe the states at the edges of the valence and conduction bands, respectively.

Upon examination of the HOMOs, we observed the two lobes of the *p*-type orbital, localized especially on O atoms, and colored red in Figure 7. Moreover, comparing the HOMOs of the three clusters, we note the high electron density strongly localized on the two central oxygen atoms, explaining the higher energy anomaly mentioned above. For the other clusters, the electron density is delocalized, with a significant contribution towards the periphery of the structure.

In contrast, the LUMOs show the four lobes characteristic for *d*-type orbitals of Ti atoms (colored grey in Figure 7). For these states, the electron density is more substantial in the central part of the structure.

At the end of this section it has to be noted that, although the HOMO–LUMO gap mimics the band gap of the semiconducting oxide, a better approximation for the gap energy, particularly when analyzing the optical properties of the system, is the electronic transition energy [17,46]. The former is determined by DFT calculations at the end of the geometry optimization, whereas the latter is computed by means of TD-DFT methods.

The optical gap is reported in Table 3. The values obtained for the optical gap range from 3.66 to 4.23 eV, systematically smaller than those for the HOMO–LUMO gap and closer to the experimental value of ∼3.4 eV, obtained with optical absorption measurements at low temperatures [75]. Although, overall, the optical gap decreases with increasing cluster size, the variation is not monotonic, the *n* = 24 anomaly being present. The values presented in Table 3 are, overall, consistent with the results of DFT calculations for stoichiometric (TiO_2_)*_n_* clusters, with *n* between 16 and 46, for which the TD-DFT gap was between 3.97 and 4.09 eV [36].

Adding to the HOMO energy, obtained by DFT, the energy of the optical transition, determined by TD-DFT, we can estimate the conduction band oxidation potentials (CBOP) (see Table 3). This energy is important for applications, to analyze the charge transfer of the adsorbed molecule to the TiO_2_ nanocluster [50,51]. The transfer can occur only if there is a proper energy level alignment, such that the excited state of the molecule lies higher than the conduction band’s oxidation potential [17,46].

### 3.2. Structure and Properties of TiO_2_ Nanoclusters with Rigid Adsorbed Molecules

In this section, we report results of calculations involving models of TiO_2_ nanoclusters and two adsorbed molecules of various sizes with a rigid backbone. In each case, we start from the optimized geometry, analyzing the binding to the cluster, and then discuss the electronic spectrum. The anchoring modes of the adsorbed molecules to the TiO_2_ surface are of crucial importance, the bonding type playing an important role in the electron injection [50,51].

#### 3.2.1. Structure and Properties of TiO_2_ Nanoclusters with a Small Rigid Adsorbed Molecule

To better understand the role of the cluster size in studying the binding of a small rigid molecule to the TiO_2_ nanocluster, we chose first the two smallest clusters (*n* = 14 and 24) and a small molecule with a rigid backbone, a coumarin-based molecule: 11-oxo-2,3,6,7-tetrahydro-1H,5H,11H-pyrano[2,3-f]pyrido[3,2,1-ij]quinoline-10-carboxylic acid (C343). The optimized geometry of C343 has been reported previously by us and other authors [52,76] and, therefore, here we only state that the structures are in agreement with the ones already presented.

**Figure 8:**
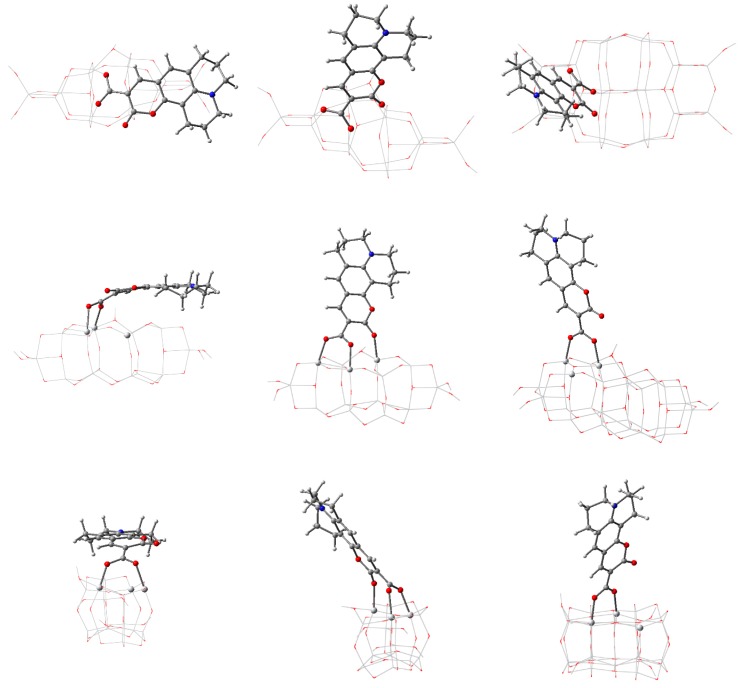
Top (**first row**), side (**second row**), and front (**third row**) views of the optimized structures of the C343 dye molecule adsorbed on the Ti_14_O_30_H_4_ cluster (**left and middle column**) and on the Ti_24_O_50_H_4_ cluster (**right column**). Calculations performed by DFT at the B3LYP/3-21G(d) level in vacuum. The side and front views also show the three neighbouring Ti atoms to which the molecule can bind. Atom colors are: C, grey; O, red; N, blue; and H, light grey.

Earlier studies have shown that for the organic dyes bearing a carboxylic acid as the anchoring group, the preferred adsorption mode is bidentate bridging, with one proton transferred to a nearby surface oxygen [25,47,77]. Here we analyze the bidentate bridge anchoring of C343 to the model clusters, with two different binding sites in the case of *n* = 14 and one binding configuration for *n* = 24. The optimized geometries are displayed in Figure 8 and some of the most important structural parameters are presented in Table 4.

The two anchoring configurations chosen for the *n* = 14 cluster connect the oxygen atoms of the adsorbed molecule to two neighboring Ti atoms on the same (101) surface of anatase titania. When the geometry optimization starts with the plane of the C343 molecule roughly transverse to the cluster’s width, the final structure exhibits the typical bidentate bridge binding, but with the adsorbed molecule strongly bent towards the cluster (left column of Figure 8). If the optimization is initiated with the C343 molecule oriented roughly along the cluster’s width, the resulting structure is highly unusual, with three Ti–O bonds (middle column of Figure 8).

Comparing geometry parameters for the isolated cluster and the two cluster-molecule systems (Table 4), we note that both the average Ti–O distances and the standard deviation of those distances increase significantly, particularly for the three-bonds case. The *n* = 14 cluster is significantly distorted in both cases, as also shown by the cluster sizes. For the two-bonds system, both the width and the length of the cluster decrease, whereas for the three-bonds model the width decreases but the length increases.

Table 4 also presents the Ti–O distances at the binding sites. The values are significantly larger than the average distance of 1.950 Å in the bulk anatase titania, suggesting that the bonds are weak, particularly for the smallest cluster. The *n* = 14 system with two bonds has highly disproportionate Ti–O distances (differences of 0.15 Å), whereas the three-bonds structure has more even distances (differences to within 0.06 Å).

On the other hand, the two-bonds structure has a smaller torsion angle of the carboxyl group relative to the coumarin plane, compared to the three-bonds system, a fact easily seen in Figure 8. This result suggests that the price paid for more even binding distances is a twisted anchor. Further, examining the dihedral angle between the molecule and the Ti atoms in the cluster, we note that the two-bonds system has the C343 dye highly tilted, almost parallel with the cluster. Of the three models analyzed, the molecule is most upright for the *n* = 24 cluster.

In contrast to the smallest cluster, the *n* = 24 system is also less distorted, a fact revealed by all key geometry parameters, starting with the average Ti–O distance and its standard deviation and continuing with the width and the length of the cluster. Moreover, the Ti–O distances at the anchoring sites differ by less than 0.03 Å, suggesting a significantly smaller distortion compared to the *n* = 14 cluster. Also, the torsion angle of the carboxyl group relative to the coumarin plane is relatively small.

To evaluate the role of the basis set on the structural parameters resulting from DFT calculations for the C343-Ti_24_O_50_H_4_ system we also used, aside from 3-21G(d), larger basis sets, LANL2DZ and a combination (DZ&6-31G) consisting of LANL2DZ on Ti and 6-31G(d,p) [78] on O, C, N, and H atoms. Table 4 shows that the average Ti–O distance increases with larger basis sets, getting closer to the value for the bulk. The difference in the length of the Ti–O bond is less than 0.22%, leading to increases in the width and the length of the cluster of about 2.8%.

Puzzled by the unusual bending, by the triple binding, and by the high distortion of the anchoring distances, we explored the energetics of the three systems. Table 5 presents the adsorption energy, determined as the difference between the calculated energy of the constituents (i.e., the oxide cluster and the C343 molecule) and the energy of the system. Binding in the two-bonds C343-Ti_14_O_30_H_4_ system is consistently the weakest, regardless of the basis set used or whether the calculation was in vacuum or in water solvent. Moreover, in one of the calculations the value is positive, indicating that the system would be better off separated. The three-bonds C343-Ti_14_O_30_H_4_ structure is also relatively weakly bound compared to C343-Ti_24_O_50_H_4_, which has the most stable structure of the three.

**Figure 9:**
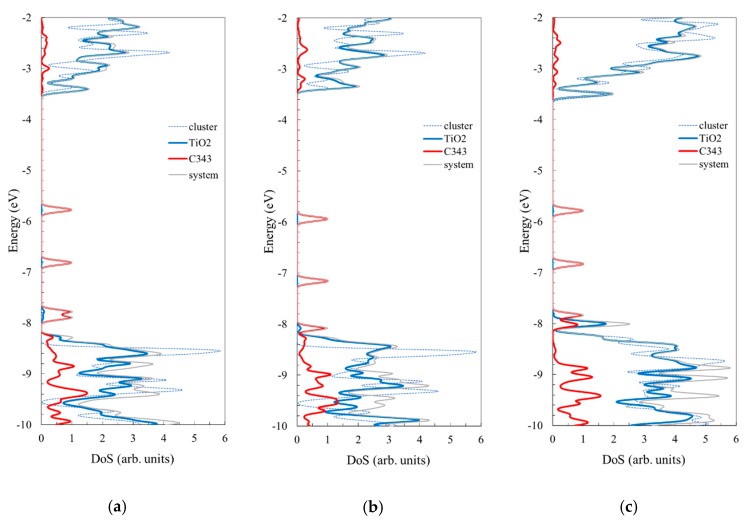
The density of states (DoS) of the systems consisting of the C343 molecule adsorbed on the Ti_14_O_30_H_4_ cluster via (**a**) two or (**b**) three O–Ti bonds and on the Ti_24_O_50_H_4_ cluster (**c**). Dotted curves represent the density of states of the corresponding bare Ti*_n_*O_2*n*+2_H_4_ clusters (*n* = 14, 24). Calculations were performed by DFT at the B3LYP/LANL2DZ level in water solvent. Energy levels were convoluted with Gaussian distributions with of 0.1 eV full width at half maximum.

Next, we examine the density of states for the three model systems considered in this section and compare them to the corresponding curves for the bare Ti*_n_*O_2*n*+2_H_4_ (*n* = 14, 24) clusters (Figure 9).

**Figure 10:**
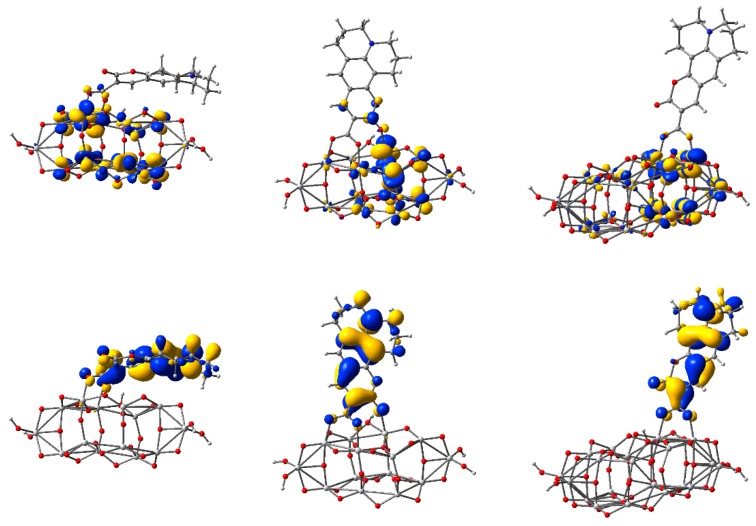
Isodensity surfaces (0.03 e/bohr^3^) of the key molecular orbitals of the systems consisting of the C343 molecule adsorbed on the Ti_14_O_30_H_4_ cluster via two (**left column**) or three (**middle column**) O–Ti bonds and on the Ti_24_O_50_H_4_ cluster (**right column**), calculated by DFT at the B3LYP/LANL2DZ level in water solvent. The first row displays the LUMOs, whereas the bottom row shows the HOMOs. Atom colors: Ti, grey; O, red; and H, light grey.

The contribution of the C343 molecule to the density of states is more significant in the valence band and particularly in the gap, where the HOMO of the complex system is located. The information provided by the density of state curves in Figure 9 is completed by the electron densities displayed in Figure 10 for the key molecular orbitals. For instance, for all three systems, the HOMO level clearly has a dominant contribution from the adsorbed molecule, the orbital having a π nature due to the conjugated character of the organic dye. In contrast, the LUMOs have the most significant contribution from the oxide, the nature of the molecular orbital being determined by the *d* atomic orbitals of Ti. However, neither the HOMOs nor the LUMOs are pure. A small weight from the cluster in the HOMOs and from the molecule in the LUMOs (contributions seen both in the density of state curves and in the electron density plots) make possible optical transitions in the visible region, as we shall see in the electronic spectra.

To examine the influence of the molecule adsorbed on the cluster on the density of states of the cluster, we compared the curves with the ones for the bare corresponding cluster (dotted lines in Figure 9). It can be seen that significant differences occur, particularly for the smallest cluster. For instance, the large peaks in the valence band at about −9.32 and −8.55 eV for the bare *n* = 14 cluster are much less intense for both types of binding and the higher one moves even higher, to −8.46 eV, for the system with three bonds. Similarly, in the conduction band, the peaks at −2.69 and −2.30 eV for the bare cluster are much less intense in the complex system. Moreover, new peaks occur at −2.96 eV. In contrast, the influence of the adsorbed molecule seems to be less significant for the *n* = 24 cluster.

By enlarging the basis set in the density of states calculations for the C343–Ti_24_O_50_H_4_ system, we found some systematic changes towards higher energies (see Figure S2 of the Supplementary Materials). The valence band edge moves up by up to 0.28 eV, the HOMO by 0.22 eV, and the conduction band edge by 0.19 eV, leading to a decrease in the band gap of 0.09 eV. We note that by using the LANL2DZ basis set on structures optimized with 3-21G(d) and LANL2DZ functions the effect on the energy of the dye states is minor.

Moving on to the electron density of the key orbitals, particularly of the ones in the conduction band, we note that despite the delocalization of the electron over the Ti atoms of the cluster, some charge is present on the atoms of the anchoring group. This fact can be seen in the LUMOs but is much more obvious for the LUMO+5 orbital of the two-bonds and the LUMO+3 of the three-bonds *n* = 14 complex system, and for the LUMO+2 of the *n* = 24 model structure (see Figure S3 of the Supplementary Materials). The presence of charge on the anchor plays an important role in charge transfer properties and has to be well-described by the structure used to model the experimental systems.

**Figure 11:**
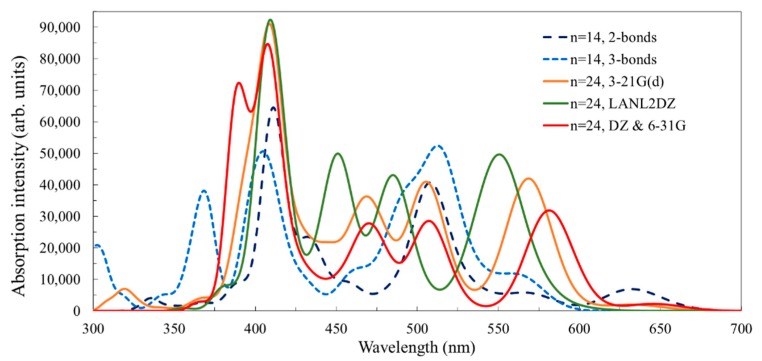
Simulated UV–Vis absorption spectra of the systems consisting of the C343 dye molecule adsorbed on the Ti*_n_*O_2*n*+2_H_4_ clusters, calculated in water solvent by TD-DFT at the B3LYP/LANL2DZ level for *n* = 14. For *n* = 24, the calculations were performed with the LANL2DZ basis set on the geometry optimized with 3-21G(d) or LANL2DZ and a combination of LANL2DZ for Ti atoms and 6-31G(d,p) for on O, C, N, and H atoms (DZ & 6-31G) on the structure determined with the same functions. The spectral lines were convoluted with Gaussian distributions of 1000 cm^−1^ full width at half maximum.

To understand the effect of the cluster size and the binding configuration on the optical properties of the system, the electronic spectra of the three structures investigated in this section are displayed in Figure 11. The spectra of the complex system of the C343 molecule with the *n* = 14 cluster exhibits some differences between the two-bonds and the three-bonds configurations. Most peaks occur together, at 575/565 nm, 508/514 nm, or 411/408 nm, for the two-bonds/three-bonds configurations of the *n* = 14 complex system. There are also two exceptions. The lowest energy transition is relatively low and occurs at 630 nm (~1.97 eV), only for the system with the two-bonds configuration, whereas a high energy transition, of higher intensity, is located at 369 nm and belongs only to the three-bonds system (involving the presence of charge density on the atoms participating in the extra bond).

When comparing the *n* = 14 with the *n* = 24 spectra, we notice the expected similarities in terms of peak positions, particularly between the systems with analogous bidentate bridge anchoring. However, the intensity is not as well-described, particularly for the 569 nm and 465 nm transitions of the *n* = 24 system, which are HOMO→LUMO+2 and HOMO→LUMO+13, respectively (see Table S1 of the Supplementary Materials).

By enlarging the basis set in the TD-DFT calculations for the C343-Ti_24_O_50_H_4_ system, we found a red shift by up to 12 nm of the high wavelength band. This tendency is consistent with the DFT density of states calculations, where we observed unequal shifts up for the HOMO and the conduction band edge, leading to a smaller energy difference between the two.

At this point, it is worth strengthening a remark made earlier, in the discussion of the density of states, regarding the low intensity of the low-energy transitions. These transitions are made possible by the fact that the HOMOs and the LUMOs are not pure but mix to different degrees small weights from the cluster in the HOMOs and from the molecule in the LUMOs. This mixture is important for the photovoltaic and photocatalytic applications.

#### 3.2.2. Structure and Properties of TiO_2_ Nanoclusters with a Large Rigid Adsorbed Molecule

Having discussed comparatively the anchoring of a small rigid molecule on two clusters of different sizes, we now move on to a larger molecule, but still rigid, to explore the influence of the size of the adsorbed molecule on the entire system. Again, we start from the optimized geometry, analysing the binding to the cluster, and then discuss the electronic spectrum and optical properties.

**Figure 12:**
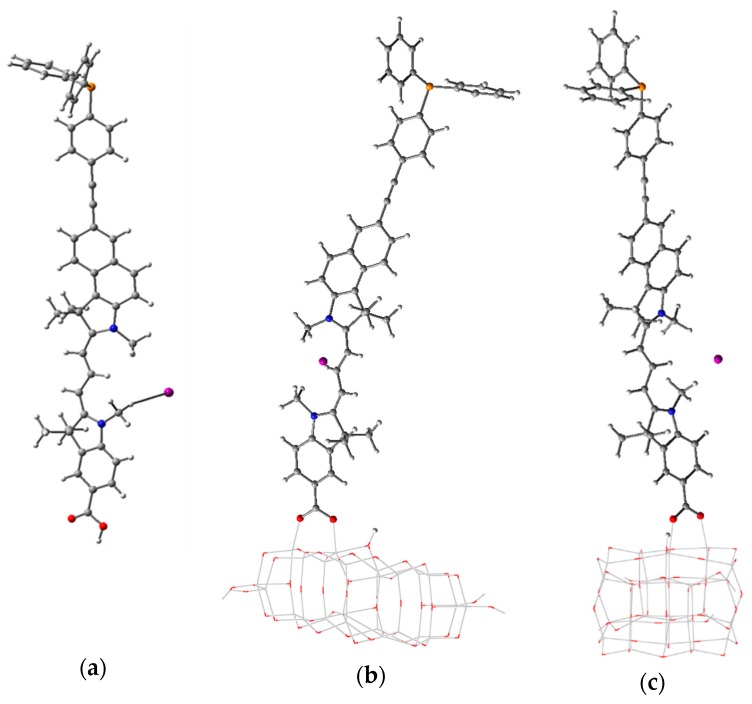
Optimized structures of the OMCD1 molecule (**a**) and the system OMCD1–Ti_24_O_50_H_4_, side view (**b**) and front view (**c**), calculated by DFT at the B3LYP/3-21G(d) level. Atom colors are: C, grey; O, red; N, blue; P, orange; I, purple; and H, light grey.

We report here results obtained for a new dye, an oligomethine cyanine molecule: 5-carboxy-2-(3-(7-((4-(diphenylphospho) phenyl)ethynyl)-1,1,3-trimethyl-1H-benzo[e] indol-2(3H)-ylidene)prop-1-en-1-yl)-1,3,3-trimethyl-3H-indol-1-ium iodide, OMCD1 [54,57] (see Figure 12). We note that the OMCD1 dye has to be a cationic species in order to ensure the π conjugation over the nitrogen atoms in the cyanine constituents. For the calculation, to ensure the overall neutrality, we placed a negative iodine ion in the vicinity of the cyanine parts, at distances of 5 Å and 5.6 Å to the N atoms. This modeling and computational artifice is particularly appropriate when modeling systems with applications in dye-sensitized solar cells, where iodine is already present in the electrolyte.

Comparing geometry parameters for the bare cluster and the cluster-molecule system (Table 6 and Figure 12), we note that the average Ti–O distances and the standard deviation of those distances are both very close, indicating only a small distortion of the cluster due to the adsorbed molecule. Similarly, the width and the length of the cluster both have a small variation from the isolated cluster, confirming the minor distortion caused by the molecule.

Table 6 also presents the Ti–O distances at the binding sites. The values are larger than the average distance of 1.950 Å in the bulk anatase titania. The two bonding Ti–O distances are relatively close, at about 0.02 Å. The torsion angle of the carboxyl group relative to the indoline ring plane is relatively small and the dihedral angle between the molecule and the Ti atoms on the surface of the cluster is close to a right angle, indicating that the molecule is upright. All parameters indicate that the adsorbed molecule, despite its size, has only a minor effect on the cluster, the distortion being small.

**Figure 13:**
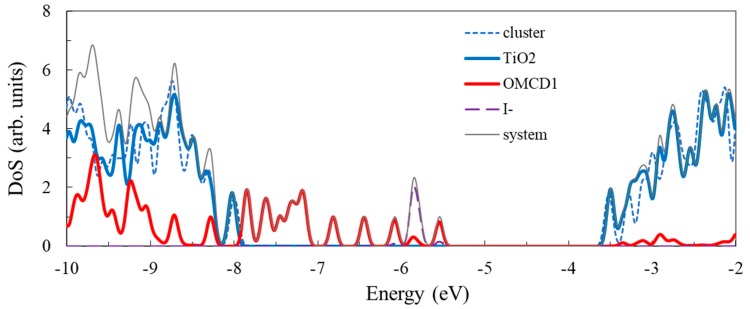
Density of states of the OMCD1–Ti_24_O_50_H_4_ system, compared to the bare Ti*_24_*O_50_H_4_ cluster, calculated by DFT at the B3LYP/LANL2DZ level in water solvent. The contributions of the various atoms are: Ti, blue; O, red; –OH groups, grey; dye molecules, black; iodine anion, green. Energy levels were convoluted with Gaussian distributions with a full width at half maximum of 0.1 eV.

The density of states for the model system consisting of the Ti_24_O_50_H_4_ clusters with the adsorbed OMCD1 molecule as well as the corresponding curve for the bare Ti_24_O_50_H_4_ cluster is displayed in Figure 13. As in the case of the smaller adsorbed molecule, presented in the previous section, the contribution of the OMCD1 molecule to the density of states is more significant in the valence band and particularly in the gap, where the HOMO of the complex system is located. The HOMO level clearly has a dominant contribution from the adsorbed molecule, the orbital having a π nature. In contrast, the LUMO has most significant contribution from the oxide, the nature of the molecular orbital being determined by the *d* atomic orbitals of Ti. We note that in this case too, the HOMO and the LUMO are not pure, a small contribution being present from the cluster in the HOMO and from the molecule in the LUMO.

We noticed in the density of states of the complex system a peak at about −5.86 eV (HOMO-3 in Figure 13), which is due to three occupied 5*p* orbitals of the negative iodine ion included in the modeling of the OMVD1 molecule. They are localized just below the HOMO and are not contributing to the regeneration process of the dye in the photovoltaic applications.

To examine the influence of the molecule adsorbed on the cluster on the density of states of the cluster, we included in the plot the curve for the bare cluster. It can be seen that some differences occur, for instance, at about −8.95 and −8.59 eV, in the valence band and at −3.20 eV in the conduction band. We note that the states at the edges of the valence and conduction bands are not influenced significantly by the presence of the adsorbed molecule.

**Figure 14:**
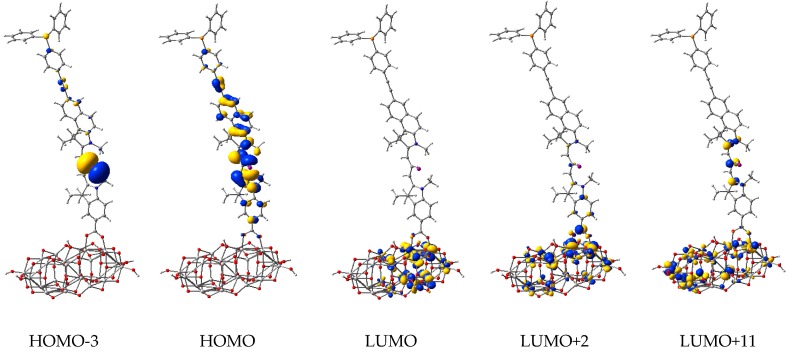
Isodensity surfaces (0.03 e/bohr^3^) of the key molecular orbitals of the system consisting of the OMCD1 molecule adsorbed onto the Ti_24_O_50_H_4_ cluster, calculated by DFT at the B3LYP/LANL2DZ level in water solvent. Atom colors: T, light grey; C, grey; O, red; N, blue; P, orange; I, purple; and H, small light grey.

The electron density of some key orbitals is displayed in Figure 14. Electron density plots are shown for HOMO-3 (at −5.86 eV), a molecular orbital with a strong contribution from the 5*p* atomic orbitals of I-, and HOMO (at −5.54 eV), corresponding to the ground state of OMCD1. From the conduction band, we represent the LUMO (at −3.50 eV), the band edge of the oxide, and two molecular orbitals with some charge density delocalized also on the adsorbed molecule, LUMO+2 (at −3.34 eV) and LUMO+11 (at −2.92 eV). These states can be readily identified in the density of states graph, the HOMO, and the excited states of OMCD1 being able to contribute to light absorption followed by a charge transfer in photovoltaic or photocatalytic applications.

### 3.3. Structure and Properties of TiO_2_ Nanoclusters with Flexible Adsorbed Molecules

In the previous section, we presented the results of a calculation involving two molecules with a rigid backbone but of different sizes adsorbed on small clusters (*n* = 14 and 24). In this section, we investigate the case of molecules with a flexible backbone and study the optimized geometry, the binding to the cluster, and the electronic spectrum. The goal is to find the optimal cluster size to model the complex system.

**Figure 15:**
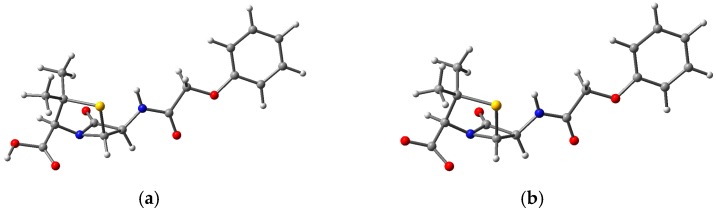
The optimized structure of (**a**) neutral and (**b**) deprotonated penicillin V calculated by DFT at the B3LYP/3-21G(d) level. Atom colors: C, grey; O, red; N, blue; S, yellow; and H, light grey.

The molecule of choice in this section is penicillin V (phenoxymethylpenicillin, 3,3-Dimethyl-7-oxo-6-(2-phenoxyacetamido)-4-thia-1-azabicyclo[3.2.0]heptane-2-carboxylic acid) (PV), a member of a group of antibiotics still widely used today in human and veterinary medicine, and present in waste water from the pharmaceutical industry and animal farms. The structures of PV, in neutral as well as in deprotonated forms, are presented in Figure 15. It was reported earlier [56] that the deprotonation of the carboxyl group has some influence on the entire structure in the case of penicillin G but less so for penicillin V. Two angles, which are of interest and will be further used, the angle of the PV relative to the carboxyl group and of the rest of the PV with respect to the phenyl group, have values of 28.5° and 1.1° for the neutral molecule and 23.5° and 1.4° for the deprotonated molecule, respectively.

**Figure 16:**
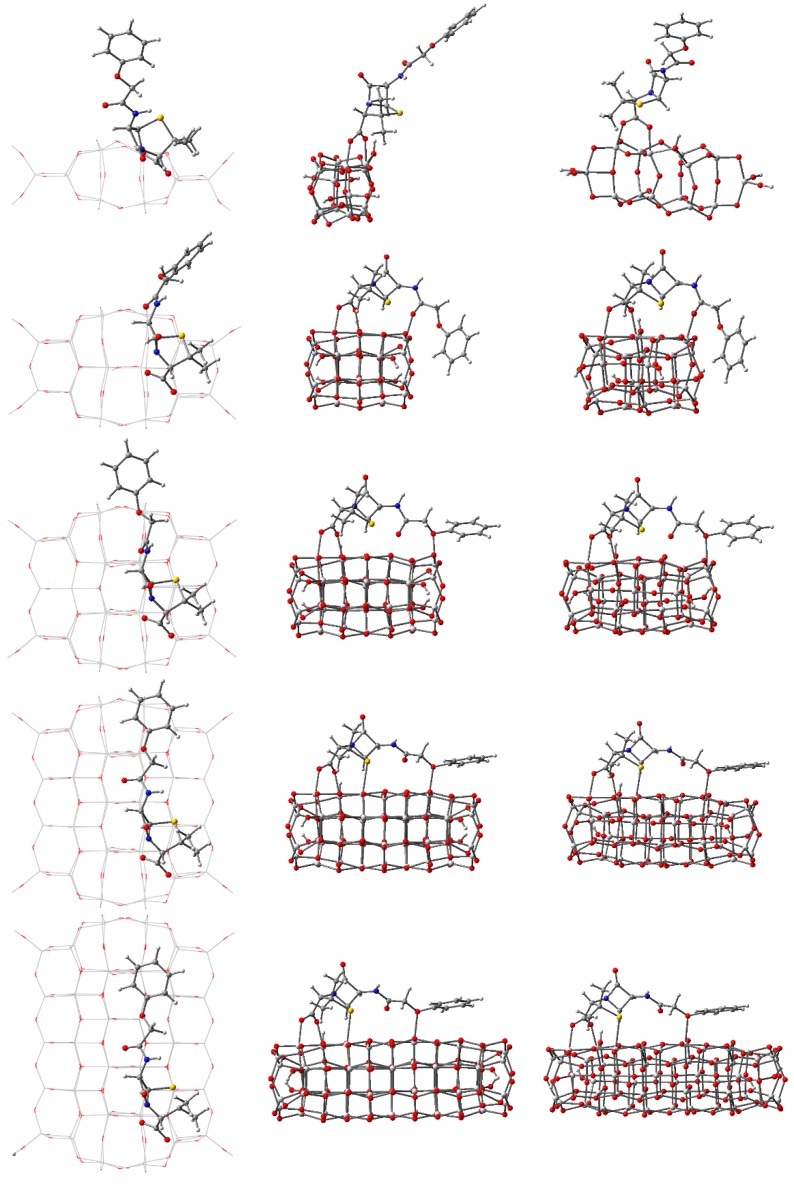
Top view (**first column**), side view (**second column**), and perspective view (**third column**) of the optimized structures of the Penicillin V molecule adsorbed on Ti*_n_*O_2*n*+2_H_4_ clusters, with *n* = 14, 24, 34, 44, 54, on the first, second, third, fourth, and fifth rows, respectively. Calculations performed by DFT at the B3LYP/3-21G(d) level in vacuum. Atom colors are: C, grey; O, red; N, blue; S, yellow; and H, light grey.

Given the known adsorption tendencies of molecules onto anatase titania [47,77], we performed DFT calculations to determine the optimized geometry of the complex systems starting from the penicillin V molecule with bidentate bridge anchoring through the carboxyl group, with the proton leaving the antibiotic and binding to an O atom of the oxide. Figure 16 and Table 7 present the optimized geometry and the most important structural parameters for the PV molecule adsorbed on all five clusters.

Examining Figure 16, the first and most important observation is that the PV molecule stands out on the smallest cluster, whereas on the other clusters it is strongly distorted, in different ways, depending on the cluster size. The energy of the total system is minimized by bending and twisting of the molecule with a flexible backbone, such that extra bonds can occur and π–π interactions [79] are made possible.

The extra bond occurs, in the case of *n* = 24, between the central O atom of the PV backbone and a Ti atom on the oxide, the peripheral phenyl group being roughly transverse to the surface. In the case of the *n* = 34 cluster, the additional bond involves the O atom connected to the phenyl group and the peripheral ring is roughly parallel to the surface. In the case of *n* = 44 and 54, there are two extra bonds to surface Ti atoms: one involves the O atom next to the phenyl ring (just as for *n* = 34) and one engaging the S atom. The tendency already observed for *n* = 34 continues, with the phenyl ring parallel to the surface, at about 2.95 Å, interacting through π–π interactions with the atoms at the surface of the oxide [79].

Comparing the data presented in Table 1 and Table 7, we observed that the presence of the adsorbed penicillin V molecule on the clusters causes some distortions to the structure. The presence of the PV causes the average Ti–O distance to be smaller by 0.65% for the *n* = 14 cluster. For the other clusters, the average distance is larger, the relative difference decreasing monotonically in absolute value with increasing *n*, from 0.43% to 0.11%. The standard deviation of the complex systems is significantly different from the values of the isolated cluster only for *n* = 14, the much wider range of distances indicating significant distortion of the structure. For the cluster width, the relative differences are less than 1% even for *n* = 14, whereas for the length the relative differences are larger than 2% for *n* = 14, 24, and 34 and smaller than 0.5% for *n* = 44 and 54.

The torsion angle between the PV backbone and the Ti–O bond is about 160° for the smallest cluster and around 130° for all others, consistent with the bending of the molecule observed in Figure 16. The angle between the carboxyl branch and the PV backbone is similar to the value for the isolated PV (28.5°) only for the smallest cluster; for the larger ones, the angle is close to 0°. At the other end of the molecule, the PV backbone suffers some mechanical stress, revealed by the torsion angle of the phenyl ring. For the smallest cluster, the angle is close to the value of the isolated PV (1.1°), whereas for the other clusters the differences are significant, going both ways.

**Figure 17:**
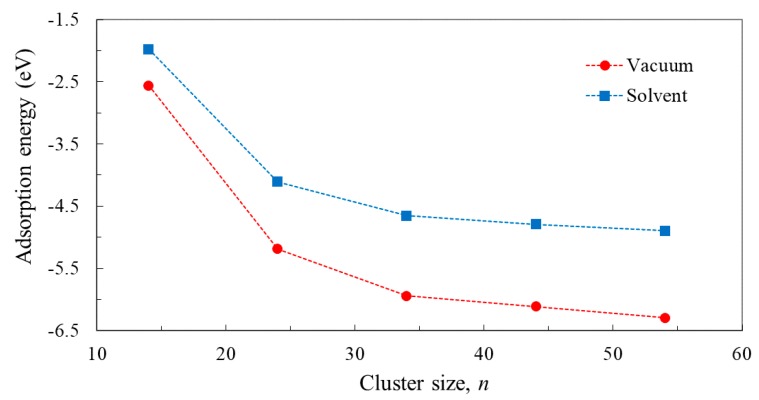
Adsorption energy of PV on the Ti*_n_*O_2*n*+2_H_4_ clusters (*n* = 14, 24, 34, 44, 54), calculated by DFT at the B3LYP/3-21G(d) level in vacuum and in water solvent.

The adsorption energy, determined as the difference between the calculated energies of the entire system and of the separated constituents, is displayed in Figure 17. We note that the binding is weak for the smaller clusters and stronger, with a tendency to saturate, for the larger clusters. This result is consistent with the extra bonds that occur when the cluster surface increases, allowing for structural relaxation.

**Figure 18:**
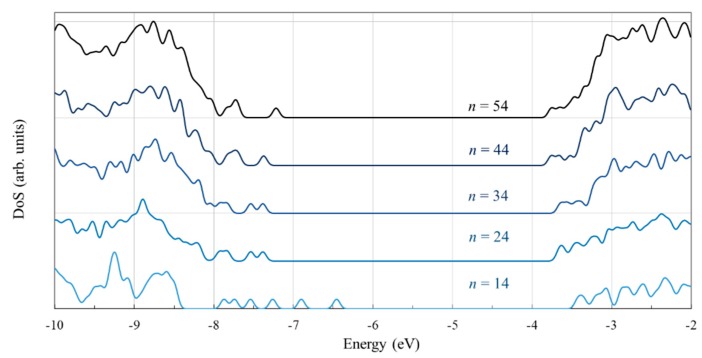
Density of states (DoS) of the systems consisting of the PV molecule adsorbed on the Ti*n*O_2*n*+2_H_4_ clusters (*n* = 14, 24, 34, 44, 54). Calculations were performed by DFT at the the B3LYP/LANL2DZ level in water solvent. Energy levels were convoluted with Gaussian distributions with a 0.1 eV full width at half maximum.

**Figure 19:**
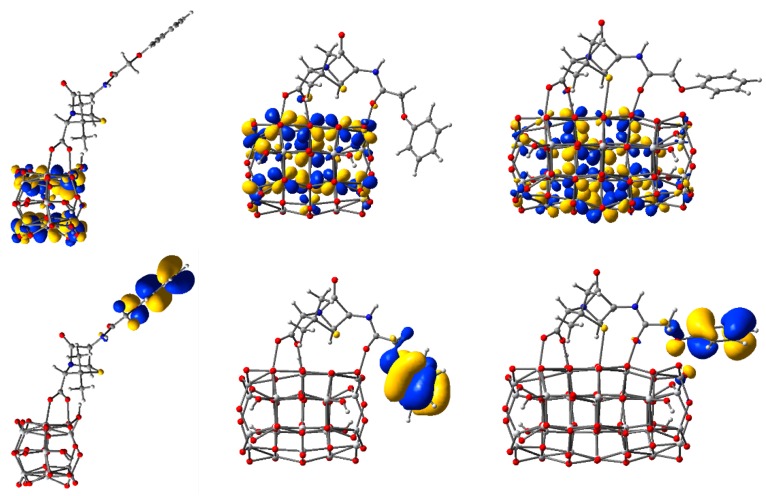
Isodensity surfaces (0.03 e/bohr^3^) of the key molecular orbitals of the systems consisting of the PV molecule adsorbed on the Ti*_n_*O_2*n*+2_H_4_ cluster (*n* = 14, **left**; *n* = 24, **middle**; and *n* = 34, **right column**), calculated by DFT at the B3LYP/LANL2DZ level in water solvent. The first row displays the LUMOs, whereas the bottom row shows the HOMOs. Atom colors: Ti, light grey; C, grey; O, red; N, blue; S, Yellow; and H, very light grey.

Next, we examined the density of states for the PV molecule adsorbed on Ti*_n_*O_2*n*+2_H_4_ to examine the role of the size of the cluster. An examination of Figure 18 reveals the valence gap located below roughly -8 eV and the conduction band above −4 eV, as seen in Figure 5 for the isolated clusters. What is new is the presence of new states in the gap that were not present in the case of the bare clusters. Both the density of states calculation and the electron density plots for the key orbitals (Figure 19) indicate that the additional states in the gap represent a contribution of the PV molecule.

In the case of the smallest cluster, when the PV molecule is only slightly distorted, the numerous such states that occur are similar to those of the isolated molecule. As the cluster size increases and the energy is lowered by extra bonding and π–π interactions, the additional states in the gap suffer significant changes in their position.

The electron density of the key molecular orbitals of the complex systems, shown in Figure 19, indicate that for all HOMOs the charge is mostly located on the phenyl group, whereas for all LUMOs the charge is distributed on Ti atoms, with a high contribution from *d* atomic orbitals.

**Figure 20:**
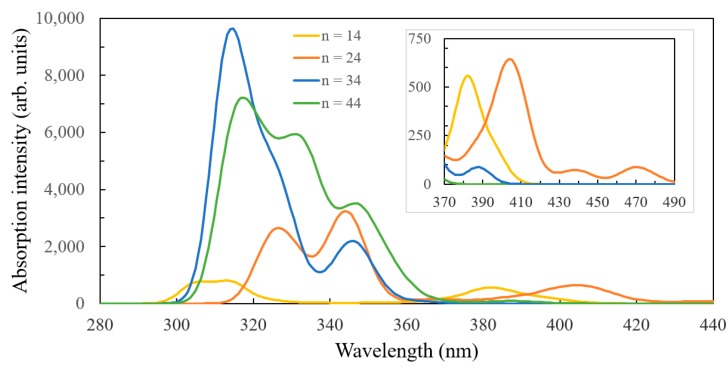
Simulated UV–Vis absorption spectra of the systems consisting of the PV molecule adsorbed on the Ti*_n_*O_2*n*+2_H_4_ clusters (*n* = 14, 24, 34, 44), calculated by TD-DFT at the B3LYP/LANL2DZ level in water solvent. The inset shows details of a higher wavelengths range. The spectral lines were convoluted with Gaussian distributions of 1000 cm^−1^ full width at half maximum.

The states in the gap having an obvious dominant PV contribution are in fact mixed, with some small weight from the oxide. This mixture allows, with a small oscillator strength, for transitions that otherwise would have been forbidden. To understand the effect of the cluster size on the optical properties of the system, the electronic absorption spectra of three complex structures are displayed in Figure 20.

The adsorbed PV molecule introduces states in the gap that move the absorption band from the UV towards the visible range of the spectrum. This behaviour is important for both photovoltaic and photocatalytic applications. A careful examination of Figure 20 reveals the presence of weak absorption bands in the blue/violet region of the spectrum, which correspond to HOMO–LUMO transitions. The positions of the optical bands, calculated with TD-DFT methods, vary significantly with the cluster size, consistent with the DFT calculations that provided the density of states.

## 4. Discussion

We are now in a position to interpret our results and discuss their relevance. We start by noting that our DFT calculations performed for the different Ti*_n_*O_2*n*+2_H_4_ clusters (*n* = 14, 24, 34, 44, 54) revealed the existence of some distortions with respect to the bulk, which decrease with the increase of *n*. As the cluster size increases the surface-to-bulk ratio decreases and, as expected, the properties of the model cluster mimic better and better those of the nanoparticles used in typical photovoltaic and photocatalytic applications (whose average size is about 20 nm [50,51], leading to over 15,000 Ti atoms [80]). Our clusters have much smaller sizes, of less than 2 nm, and were intentionally constructed to model interface phenomena, which involve the anatase titania (101) surface to be well-approximated. That is why, when enlarging our clusters, we did not add layers in depth, which would have increased the number of 6-fold coordinated Ti atoms, and decreased the surface-to-bulk ratio. On the contrary, we enlarged the surface to better describe the interface effects, a fact that proved to be crucial in the proper treatment of flexible adsorbed molecules, such as PV.

Our results for the cluster structures compare well, particularly with those obtained by Persson et al. [33,34,35,36,37,38,39], who also used DFT, and to some extent with those of De Angelis et al. [41,42,43,44,45,46,47], who obtained the geometry by molecular dynamics simulations. More recently, Chen and Dixon [81] used a hybrid generic algorithm to determine the equilibrium structure of ultra-small nanoparticles of TiO_2_, with *n* from 1 to 384, and for *n* up to 80 the geometry optimization being finalized with DFT at the B3LYP/DZVP2 level. They report similar tendencies of the TiO_2_ nanoclusters to display distortions from the bulk, particularly for clusters of smaller sizes. We note that our *n* = 14 and 24 clusters have a similar structure with their 14a and 24b clusters; for the other sizes, the numbers of Ti atoms are different, similarities being identified between our *n* = 34 and their 36b as well as our *n* = 44 and their 48a [82]. Moreover, consistent with our results, their study demonstrated that the band gap of the ultra-small nanoparticles is strongly affected by the particle shape and surface structure, the gap decreasing with cluster size (scaling with *n*^−1/2^).

Our calculations indicated, through the tendency to saturate of (*i*) the surface-to-bulk ratio, (*ii*) the binding energy per atom, (*iii*) the DFT calculated HOMO–LUMO band gap as well as (*iv*) the TD-DFT determined optical gap, that the cluster with *n* = 54 brings little improvement compared to the one with *n* = 44, but increases significantly the computational cost. Our findings are consistent with previous reports, particularly those of Persson and co-workers, who used clusters with *n* = 38 [35] or 46 [37] and, later, 92 [39], and De Angelis and co-workers, who employed clusters with *n* = 38 [43] and with *n* = 82 [44].

Jug and co-workers used free and hydrogen-saturated clusters for the simulation of anatase surfaces by means of semi-empirical methods and found that the influence of the cluster size on the convergence of the adsorption energies in the series *n* = 33, 56, 72, 132 was insignificant [40]. Our DFT calculations, performed on clusters saturated with four peripheral –OH groups, suggest that these groups play a minor role in the density of states of the clusters and, in any case, not for the states that are crucial for photovoltaic or photocatalytic applications. Studying clusters with *n* = 29 70, and 121, Vorontsov [82,83] used the self-consistent-charge density-functional tight-binding method (SCC-DFTB) and found, on one hand, that the energy of the HOMO and especially the LUMO substantially depends on the location of the surface hydroxyl groups and, on the other hand, that the band gap is underestimated with respect to the experimental value [50].

The study of rigid adsorbed molecules allowed for a double analysis with respect to the size of the cluster as well as the size of the molecule. The anchoring of the C343 molecule on the *n* = 14 cluster revealed significant distortions of both the cluster and the molecule, with highly either uneven Ti–O bond lengths for the bidentate bridge anchoring, associated with strong bending of the molecule towards the surface or with unusual tridentate binding configurations. Such unusual behavior is not seen for larger clusters, not even for *n* = 24, as for such structures the adsorbed molecule tends to stand upright, almost perpendicular to the oxide surface. The adsorption energy comparisons showed significantly weaker bonding for *n* = 14 with respect to *n* = 24, the increase in size leading to more stable structures. When contrasting the optical spectra, we noticed some similarities in terms of peak positions, particularly between the systems with analogous bidentate bridge anchoring. However, the intensity was not as well-described, particularly for the higher wavelength transitions occurring in the visible range of greater interest for photovoltaic and photocatalytic applications.

As our results contradict those of several previous studies, which used very small clusters, we argue that the TiO_6_H_5_ complex used by other authors [23,24] cannot be ‘sufficient for the investigation of geometries, excitations, and electronic structures of the as-investigated dyes before and after binding’ and should be used with precautions. One obvious reason is that such a complex could only consider bidentate chelate anchoring, in contrast with the vast experimental and computational data, indicating bidentate bridging [25]. Moreover, we warn that small clusters, such as Ti_5_O_20_H_22_ [26], (TiO_2_)_6_ [27,29,30], Ti_6_O_21_H_18_ [28] (TiO_2_)_9_ [31,84], and even (TiO_2_)_15_ [32] or (TiO_2_)_16_ [85], may not reproduce sufficiently well the electronic structure and optical spectra of the small molecules bound to the substrate.

When attaching the larger but still rigid OMCD1 molecule onto the *n* = 24 cluster, we observed relatively small distortions of either the cluster or the molecule. The binding configuration was the expected bidentate bridge, with the adsorbed molecule almost perpendicular to the surface. The density of states showed that, although the large molecule adsorbed on the relatively small cluster affected some of the states in the valence and conduction bands, the key orbitals at the edges of the bands were not altered significantly.

The adsorption of a molecule with a flexible backbone on the different clusters showed important differences, which cannot be ignored. The anchoring of the PV molecule on the *n* = 14 cluster left the molecule almost unaffected. However, that situation proved to be deceiving, as attaching the molecule onto increasingly larger clusters demonstrated that the energy of the system can be lowered substantially by distorting the PV. The bending and twisting of the backbone allowed for energy minimization due to extra bonds and π–π interactions. The tendency to saturate of the adsorption energy indicated that the benefits brought about by the cluster with *n* = 54 are not significant compared to the one with *n* = 44. The same tendency to saturate comes from the analysis of the density of states, obtained by DFT calculations, and of the electronic absorption spectra, determined by TD-DFT methods. Our results compare well with the pioneering work of Persson et al. [36,37] and De Angelis et al. [41,44], as well as with more recent reports [86,87,88,89], which use clusters with an *n* of at least 38.

Now, to link the results of our calculations, summarized above, to practical applications, such as in hybrid photovoltaics or in photocatalytic degradation of pollutants under visible light, we need to address two key processes: light absorption and charge transfer from the molecule to the oxide. For that reason, it is useful to recall the typical approach used to investigate whether a molecule is a good candidate as a dye to sensitize TiO_2_ in dye-sensitized solar cells (DSSC) [50,51]. One condition that a sensitizer has to fulfil is the proper anchoring to the oxide, which is considered a precondition for a high rate of charge transfer. The –COOH group at the periphery of a dye has the right positions of the atoms and the right symmetry of the molecular orbital to ensure both a strong mechanical binding but also a high overlap of the *p* orbitals of O and the *d* orbitals of Ti, for a sizeable electron transfer probability.

A second criterion is the matching of the absorption spectrum of the dye with the solar irradiation spectrum [51]. Our results showed that the optical spectra, provided by TD-DFT calculations for clusters of various sizes, indicate significant changes in both wavelength and intensity of the electronic transitions in the visible range, where the solar intensity reaches its maximum. Moreover, we found that the spectra were generally insufficiently described by the small clusters, sizes of *n* = 34 and 44 being sufficient for most practical purposes (given the molecules studied here), whereas *n* = 54 brought little further improvement.

Another condition for the dye is the charge injection into the oxide. The high rate of charge injection for typical organic sensitizers is achieved if the molecule exhibits the “push→pull” effect [51]. This effect is seen in dyes with an electron-rich donor part at one end and an acceptor part at the opposite side, ending in an anchoring group (such as the carboxyl group), and kept together by a π bridge. The key for charge injection is the alternant single/double bond character of the molecular backbone, which allows for electron delocalization. A push-pull effect occurs when the ground state of the dye has the charge localized on the donor part and the excited state, reached after light absorption, has the charge localized on the acceptor part, right next to the TiO_2_. The push→pull effect can be studied by analyzing how the electron density is localized in the ground and excited states of the dye adsorbed on the cluster. A proper description of the charge delocalization is crucial for this purpose, which, as our results demonstrated, requires a suitable account of the structure, by an adequate choice of the TiO_2_ cluster.

The charge injection also depends on the proper alignment of the excited state of the dye with respect to the conduction band edge of the oxide. Additionally, another criterion is the dye regeneration, by returning the electron to the dye after passing through and performing work in the external circuit. In DSSCs, this process is performed by the electrolyte (or, equivalently, by a hole conductor) if the redox level of the electrolyte is situated above the ground state of the dye. The calculations leading to the density of states reveal the position of the ground state and the excited state resulting after light absorption, allowing for the analysis of the energy level alignment. Moreover, the use of TD-DFT calculations providing the optical gaps lead to the conduction band oxidation potential (see Figure S5 of the Supplementary materials), which can be used as an alternate to the conduction band edge in the discussion of the alignment.

Our results show that both the conduction band edge and the ground state of the dye are influenced, to various extents, by the size of the cluster. The differences can be observed from the density of states calculations for the C343-Ti*_n_*O_2*n*+2_H_4_ systems, where they are small but significant, but more so for the PV-Ti*_n_*O_2*n*+2_H_4_ structures, where they become important. Under these circumstances, the influence of the cluster size on the proper description of the energy level alignment has to be taken into account when choosing the cluster size used in the simulation.

As a final point, Illas and co-workers [80] showed that the exchange contribution to the hybrid DFT functional strongly affects the calculated bang gap of TiO_2_. We performed calculations of the C343-Ti_24_O_50_H_4_ system with the B3LYP functional and various basis sets for determining the optimized geometry, the electronic structure, and the UV–Vis spectra. Our calculations are consistent with the early work of Persson and co-workers [36], emphasizing the need for consistency in computations.

## 5. Conclusions

We reported DFT calculations of several TiO_2_ clusters and complex systems consisting of the cluster with an adsorbed molecule to study the influence of the cluster size on the electrical and optical properties of materials/interfaces, having in mind hybrid photovoltaic and photocatalytic applications.

We performed calculations for different Ti*_n_*O_2*n*+2_H_4_ clusters (*n* = 14, 24, 34, 44, 54), which revealed the existence of some distortions with respect to the bulk, which are larger for smaller clusters. The tendency of the cluster with n = 54 to saturate the surface-to-bulk ratio, the binding energy per atom, the electronic (DFT) band gap, as well as the optical (TD-DFT) gap brings about little improvement compared to the one with n = 44, but increases significantly the computational cost.

The study of the complex systems, consisting of the cluster with the adsorbed molecule, demonstrated for the *n* = 14 case significant distortions of both the cluster and the molecule and unusual tridentate binding configurations. Such behaviour is not seen for larger clusters, not even for *n* = 24. The adsorption energy comparisons showed for *n* = 14 significantly weaker bonding, differences in the density of states (particularly the positions of the key orbitals and their relative intensity), and in the optical spectra (both in terms of peak wavelength and intensity). Under these circumstances, we conclude that the *n* = 14 cluster is a poor choice for simulating the materials used in the practical applications envisaged here.

The use of the *n* = 24 cluster has provided mixed results. First, the inner symmetry of the cluster structure causes stronger electron localization of the electron density at the centre of the cluster, causing the HOMO to move higher in energy and split away from the immediately lower energy states. This anomaly leads to a smaller DFT band gap and TD-DFT optical gap. The cluster showed some distortions but described qualitatively well the binding of the rigid molecules, even for those of a larger size. For flexible molecules, the *n* = 24 cluster indicated correctly the bending and twisting of the adsorbed molecule but, due to its small size, was not able to describe the correct geometry and the π–π interactions. Consequently, we conclude that the *n* = 24 cluster can be used well for qualitative calculations, particularly when the adsorbed molecule is rigid, or to obtain some initial information to set up further more elaborate, computational experiments. However, if accuracy is important or the molecule is flexible, the structure is unlikely to provide the precision required by such applications as hybrid photovoltaics or photocatalytic degradation of pollutants under visible light.

When the molecule is adsorbed on the larger clusters, starting from *n* = 34, the structure and the electronic and optical properties are described in a more reliable way.

We reviewed the important criteria used in computational studies to explain or predict the suitability of various dyes as TiO_2_ sensitizers in DSSCs or the likelihood of the degradation of organic pollutants under visible light irradiation using anatase titania as a photocatalyst. We emphasized the influence of the cluster size on the proper description of the anchoring to the oxide, ruling out the *n* = 14 cluster for all systems and using with high caution the *n* = 24 cluster when molecules are flexible.

The analysis of the matching between the absorption spectrum of the dye with the solar irradiation spectrum relies on quality UV–Vis simulated spectra. Our results showed significant changes in both wavelength and intensity of the electronic transitions in the visible range, the spectra being qualitatively described by the *n* = 24 cluster, and more accurate results being obtained for larger systems.

The charge injection into the oxide is influenced by the anchoring, by the push→pull effects determined by the charge distribution of the ground and excited states of the dye, and by the energy level alignment between the excited state of the dye and the conduction band edge of the oxide. Hence, the accuracy of the geometry optimization, of the density of states, and of the optical spectrum calculations are very important. Such accuracy is obtained with larger clusters.

The dye regeneration, by passing the electron from the electrolyte back to the dye, can happen only if the redox level of the electrolyte lies higher than the ground state of the dye. A proper approach to check this criterion requires a reliable calculation of the density of states, which, again, is high for larger clusters.

In summary, our results lead us to the conclusion that cluster sizes larger than or equal to *n* = 34 are necessary to provide the reliability required by photovoltaic and photocatalytic applications. The tendency to saturation of the key quantities of interest when moving from *n* = 44 to *n* = 54 suggests that the largest cluster may bring little improvement at a significantly higher computational cost. Probably, for many molecules of practical interest, sizes of *n* = 34 and particularly *n* = 44 may be an appropriate choice.

Finally, an important word of caution is mandatory. Consistency in calculations may be an even more important rule in the simulation of practical systems, as the functionals and the basis sets used in the DFT and TD-DFT computations may have an even stronger influence on the final results than the size of the TiO_2_ cluster.

## Figures and Tables

**Table 1:** Structural parameters describing the optimized geometry of Ti*_n_*O_2*n*+2_H_4_ clusters (*n* = 14, 24, 34, 44, 54) performed by DFT at the B3LYP/3-21G(d) level in vacuum. The average Ti–O distance and its standard deviation, and the cluster width and length, defined by the positions of the peripheral Ti or O atoms and determined by calculations are compared to the experimental values of the bulk TiO_2_ [72]. All quantities are expressed in Å.

Parameter	Ti_14_O_30_H_4_	Ti_24_O_50_H_4_	Ti_34_O_70_H_4_	Ti_44_O_90_H_4_	Ti_54_O_110_H_4_	TiO_2_
*r*(Ti–O)	1.834	1.876	1.889	1.896	1.901	1.950
*σ_r_*_(Ti–O)_	0.074	0.100	0.100	0.103	0.105	0.022
Width (Ti…Ti)	12.45	12.18	11.87	11.94	12.00	12.04
Width (O…O)	-	13.28	13.36	13.28	13.29	12.83
Length (Ti…Ti)	3.55	7.20	10.67	14.47	18.05	
Length (O…O)	4.94	8.16	12.05	15.63	18.76	
Length bulk (Ti…Ti)	3.78	7.59	11.37	15.14	18.92	

**Table 2:** The number of Ti or O atoms *m*-fold coordinated (*m*x) to O or Ti atoms, respectively, and the surface-to-bulk ratio (s/b, determined as the fraction of under-coordinated atoms [36]).

Parameter	Ti_14_O_30_H_4_	Ti_24_O_50_H_4_	Ti_34_O_70_H_4_	Ti_44_O_90_H_4_	Ti_54_O_110_H_4_	TiO_2_ (Bulk)
Ti, 6x	0	2	4	6	8	all Ti atoms
Ti, 5x	6	14	22	30	38	0
Ti, 4x	8	8	8	8	8	0
O, 3x	6	18	30	42	54	all O atoms
O, 2x	20	28	36	44	52	0
O, 1x	4	4	4	4	4	0
s/b (%)	86.4	73.0	67.3	64.2	62.2	0

**Table 3:** Valence (*E*_VB_) and conduction (*E*_CB_) band edges, the band gap (*E***_CB_** − *E***_VB_**), the optical gap, and the conduction band oxidation potentials (CBOP) (in eV), calculated for clusters of various sizes, using DFT and Time-Dependent DFT (TD-DFT), the B3LYP functional, LANL2DZ basis sets, and water solvent.

Cluster	*E*_VB_ HOMO	*E*_CB_ LUMO	*E*_CB_ − *E*_VB_ HL Gap	*E*_0-0_ Optical Gap	CBOP *E*_VB_ + *E*_0-0_
Ti_14_O_30_H_4_	−8.52	−3.37	5.15	4.23	−4.29
Ti_24_O_50_H_4_	−7.97	−3.53	4.44	3.66	−4.31
Ti_34_O_70_H_4_	−8.21	−3.70	4.52	3.81	−4.41
Ti_44_O_90_H_4_	−8.17	−3.77	4.40	3.75	−4.42
Ti_54_O_110_H_4_	−8.17	−3.79	4.38	3.75	−4.42

HOMO, highest occupied molecular orbital; LUMO, lowest unoccupied molecular orbital.

**Table 4:** The structural parameters of the model system consisting of the Ti*_n_*O_2*n*+2_H_4_ clusters (*n* = 14, 24) with an adsorbed C343 molecule, obtained after geometry optimization by DFT with the B3LYP functional, in vacuum. The basis sets used were 3-21G(d) for *n* = 14 and 3-21G(d), LANL2DZ, and a combination of LANL2DZ on Ti and 6-31G(d,p) on O, C, N, and H atoms (DZ&6-31G) for *n* = 24. Geometrical parameters describing the adsorption modes: bond lengths (in Å), torsion angle of the carboxyl group relative to the coumarin plane, and dihedral angle defined by the coumarin plane and the plane of the three neighbour Ti atoms (both in deg).

Parameter	C343 on Ti_14_O_30_H_4_ (Two Bonds)	C343 on Ti_14_O_30_H_4_ (Three Bonds)	C343 on Ti_24_O_50_H_4_		
			3-21G(d)	LANL2DZ	DZ&6-31G ^†^
*r*(Ti–O)	1.852	1.880	1.887	1.891	1.891
*σ_r_*_(Ti–O)_	0.090	0.119	0.108	0.121	0.116
Width (Ti…Ti)	12.41	12.07	11.98	12.44	12.23
Width (O…O)	-	-	13.13	13.41	13.49
Length (Ti…Ti)	3.48	3.75	7.25	7.26	7.30
Length (O…O)	5.09	5.64	8.28	8.36	8.35
*r*(O1–Ti)	2.128	2.068	1.972	1.951	1.956
*r*(O2–Ti)	1.976	2.039	1.999	2.053	2.058
*r*(O3–Ti)	-	2.096	-	-	-
<(O–C–C–C)	2.0	31.8	−4.3	0.2	−1.3
<(C343;3Ti)	8.3	52.2	81.7	88.8	85.8

^†^ combination of LANL2DZ on Ti and 6-31G(d,p) on O, C, N, and H atoms.

**Table 5:** The adsorption energy of the C343-Ti*_n_*O_2*n*+2_H_4_ systems (*n* = 14, 24), defined as the difference between the calculated energy of the bound system and the sum of the energies for the separated constituents (isolated Ti_2*n*_O*_n_*_+2_H_4_ and C343), (in eV), calculated by the DFT/B3LYP method with 3-21G(d) or LANL2DZ basis sets, in vacuum or in water solvent.

Method	C343-Ti_14_O_30_H_4_, Two-Bonds	C343-Ti_14_O_30_H_4_, Three-Bonds	C343-Ti_24_O_50_H_4_
B3LYP/3-21G(d) vacuum	−2.473	−3.075	−3.254
B3LYP/3-21G(d) solvent	−1.961	−2.574	−2.835
B3LYP/LANL2DZ solvent	0.429	−0.242	−1.188

**Table 6:** The structural parameters of the model system consisting of the Ti_24_O_50_H_4_ clusters with an adsorbed OMCD1 molecule, and of the bare cluster, obtained after geometry optimization by DFT/B3LYP/3-21G(d) in vacuum. Geometrical parameters describing the adsorption modes: bond lengths (in Å), the torsion angle of the carboxyl group relative to the indoline plane, and the dihedral angle defined by the indoline plane and the plane of the three neighbor Ti atoms on the cluster surface (both in deg).

Parameter	OMCD1 on Ti_24_O_50_H_4_	Ti_24_O_50_H_4_
*r*(Ti–O)	1.880	1.876
*σ_r_*_(Ti–O)_	0.102	0.100
Width (Ti…Ti)	12.20	12.18
Width (O…O)	13.22	13.28
Length (Ti…Ti)	7.21	7.20
Length (O…O)	8.15	8.16
*r*(O1–Ti)	2.012	-
*r*(O2–Ti)	1.989	-
<(O–C–C–C)	−4.2	-
<(Phenyl;3Ti)	87.2	-

**Table 7:** The structural parameters of the model system consisting of the Ti*_n_*O_2*n*+2_H_4_ clusters (*n* = 14, 24) with an adsorbed penicillin V molecule, obtained after geometry optimization by DFT/B3LYP/3-21G(d) in vacuum. Geometrical parameters describing the adsorption configurations: bond lengths (in Å) and torsion angles (in deg.) of the PV relative to the Ti–O bond, to the carboxyl group, and of the rest of the PV with respect to the phenyl group ^1^.

Parameter	PV on Ti_14_O_30_H_4_	PV on Ti_24_O_50_H_4_	PV on Ti_34_O_70_H_4_	PV on Ti_44_O_90_H_4_	PV on Ti_54_O_110_H_4_
*r*(Ti–O)	1.822	1.884	1.896	1.900	1.903
*σ_r_*_(Ti–O)_	0.180	0.104	0.104	0.103	0.107
Width (Ti…Ti)	12.34	12.21	11.87	11.95	12.01
Width (O…O)		13.27	13.34	13.35	13.32
Length (Ti…Ti)	3.63	7.40	10.90	14.53	18.11
Length (O…O)	4.81	8.82	11.64	15.30	18.80
*r*(O1–Ti)	2.040	2.073	2.077	2.041	2.044
*r*(O2–Ti)	2.035	2.102	2.100	2.119	2.110
<(Ti–O–C–C)	159.4	129.1	129.7	130.0	130.4
<(O–C–C–N)	25.7	0.8	1.0	−0.8	−0.5
<(C–C–O–C)	0.6	−30.4	79.9	47.4	42.3

^1^ See Figure S4 in the Supplementary Materials for details.

## Supplementary Materials

The following are available online at https://www.mdpi.com/2079-4991/9/3/357/s1, Figure S1: a Ti_54_O_110_H_4_ nanocluster illustrating how the widths and the lengths reported in Table 1 were measured, Figure S2: Density of states of the C343-Ti_24_O_50_H_4_ system calculated by DFT using the B3LYP functional and three different basis sets, Figure S3: Electron density of key orbitals C343-Ti*_n_*O_2*n*+2_H_4_ (*n* = 14, 24), Table S1: Energy, wavelength, oscillator strength and composition of the most intense optical transition for C343-Ti*_n_*O_2*n*+2_H_4_ clusters (*n* = 14, 24), Figure S4: Structure of PV-Ti_14_O_30_H_4_ to illustrate the geometrical parameters mentioned in Table 7, Figure S5: Energy level alignment for Ti*_n_*O_2*n*+2_H_4_ (*n* = 14, 24, 34, 44, 54).
