# Supplementary material for: Structure and Electronic Properties of TiO2 Nanoclusters and Dye–Nanocluster Systems Appropriate to Model Hybrid Photovoltaic or Photocatalytic Applications
Source: Nanomaterials (Basel). 2019 Mar 4;9(3):357. doi: 10.3390/nano9030357 (PMC6474027; doi:10.3390/nano9030357)
Supplement: Supplementary file 1 [file nanomaterials-09-00357-s001.pdf]

nanomaterials

Supplementary Materials

Structure and Electronic Properties of TiO_2 Nanoclusters and Dye–Nanocluster Systems Appropriate to Model Hybrid Photovoltaic or Photocatalytic Applications

Corneliu I. Oprea and Mihai A. Gîrțu *

Department of Physics and Electronics, Ovidius University of Constanța, Constanța 900527, Romania; cornel.oprea@univ-ovidius.ro

* Correspondence: mihai.girtu@univ-ovidius.ro

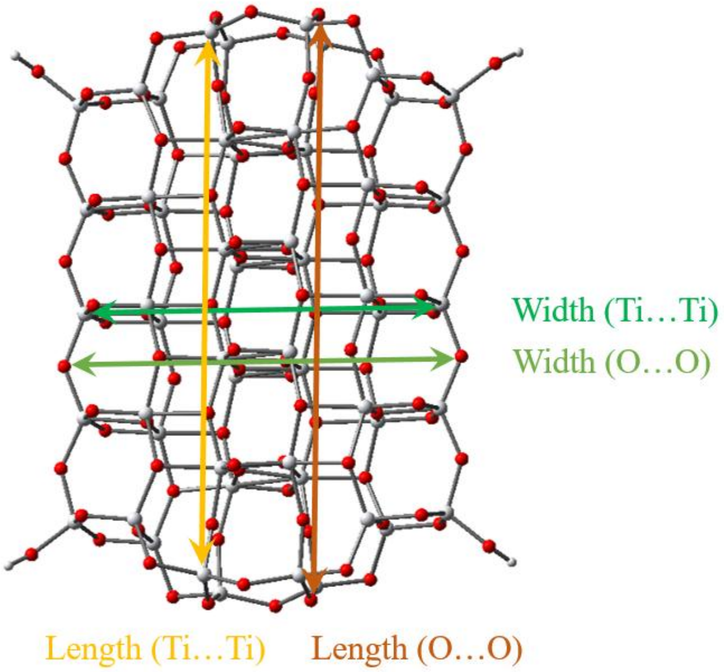

Figure S1. Top view of the optimized structures of the $Ti_{54}O_{110}H_4$ nanocluster, modelling the anatase titania (101) surface to illustrate how the widths and the lengths reported in Table 1 were measured.

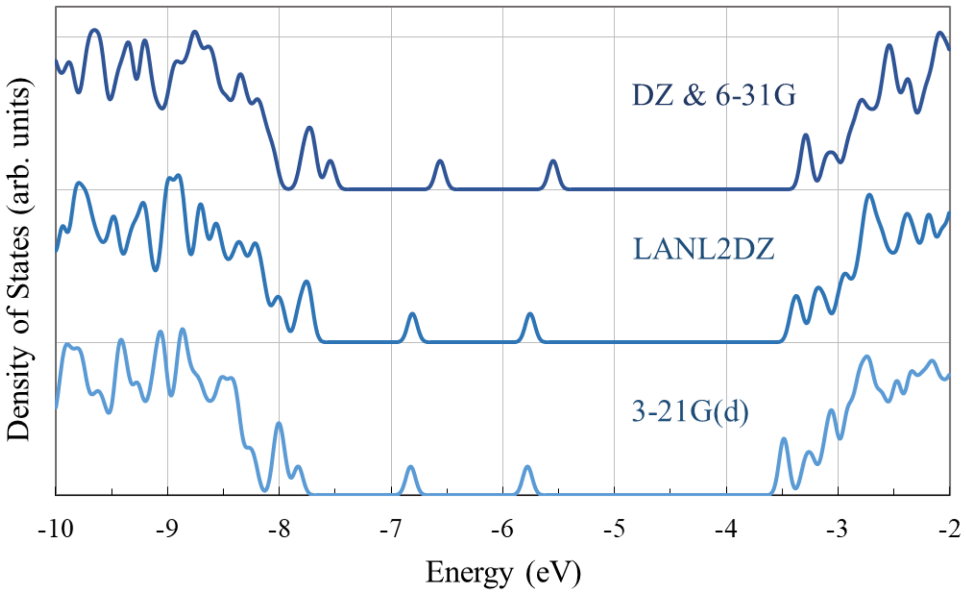

Figure S2. Density of states of the C343-$Ti_{24}O_{50}H_4$ system calculated by DFT using the B3LYP functional and three different basis sets: 3-21G(d), LANL2DZ and a combination (DZ&6-31G) consisting of LANL2DZ on Ti and 6-31G(d,p) on O, C, N, and H atoms. Energy levels were convoluted with Gaussian distributions with of 0.1 eV full width at half maximum.

State	C343-$Ti_{14}O_{30}H_4$, 2 bonds Energy (eV)	C343-$Ti_{14}O_{30}H_4$, 3 bonds Energy (eV)	C343-$Ti_{24}O_{50}H_4$, Energy (eV)
LUMO+6	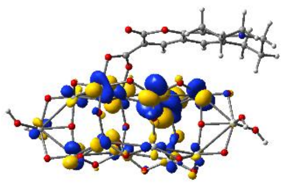 −2.923	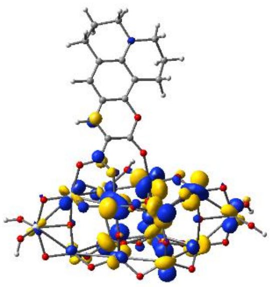 −2.945	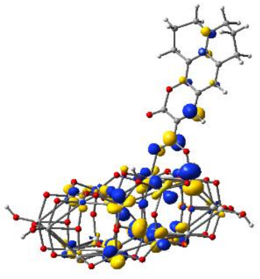 −3.052
LUMO+5	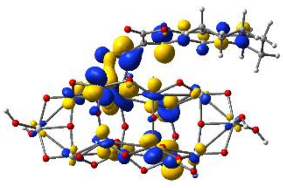 −2.989	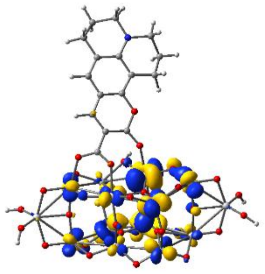 −2.974	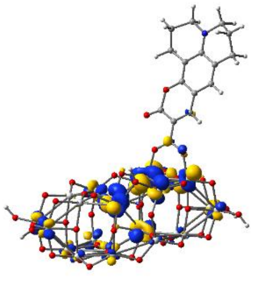 −3.083
LUMO+4	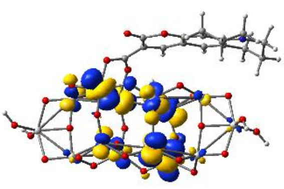 −3.029	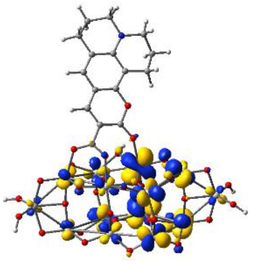 −3.059	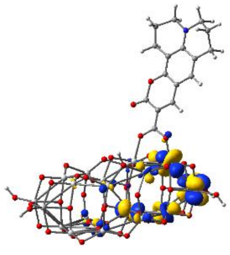 −3.131

LUMO+3	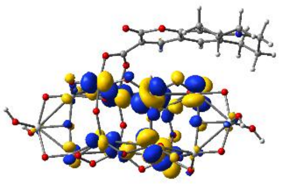 −3.090	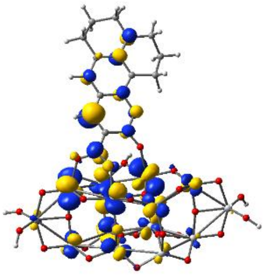 −3.181	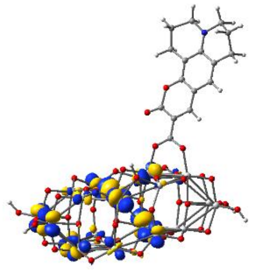 −3.233
LUMO+2	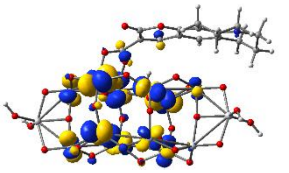 3.188	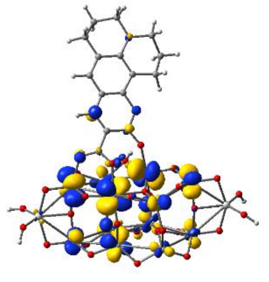 −3.252	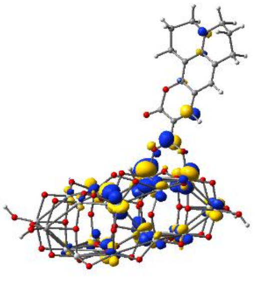 −3.297
LUMO+1	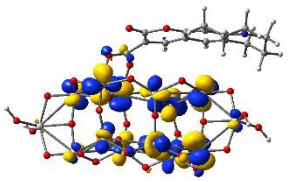 −3.365	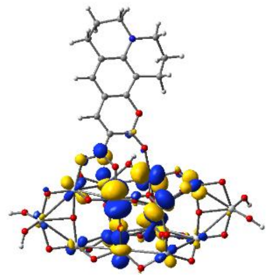 −3.329	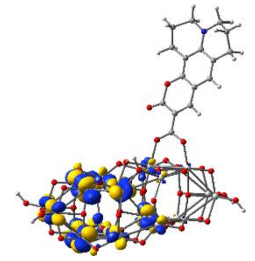 −3.481
LUMO	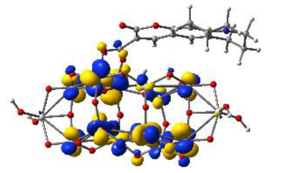 −3.425	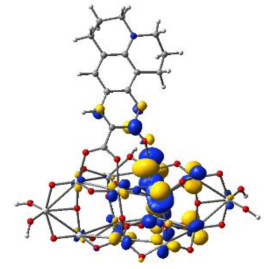 −3.354	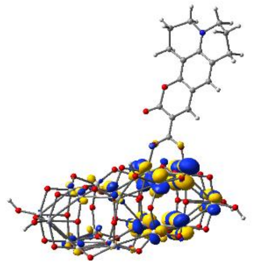 −3.494
HOMO	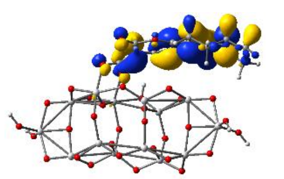 −5.771	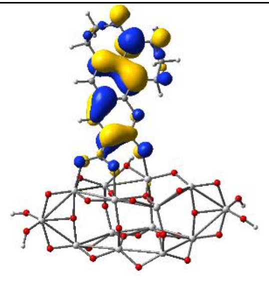 −5.934	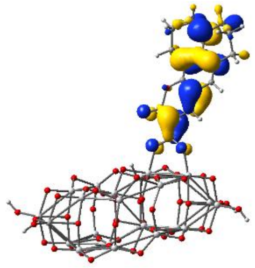 −5.781
HOMO-1	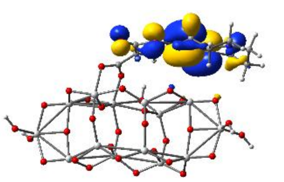 −6.812	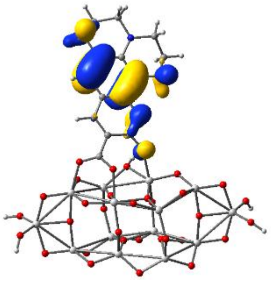 −7.153	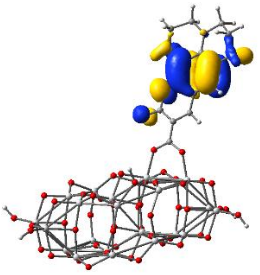 −6.827

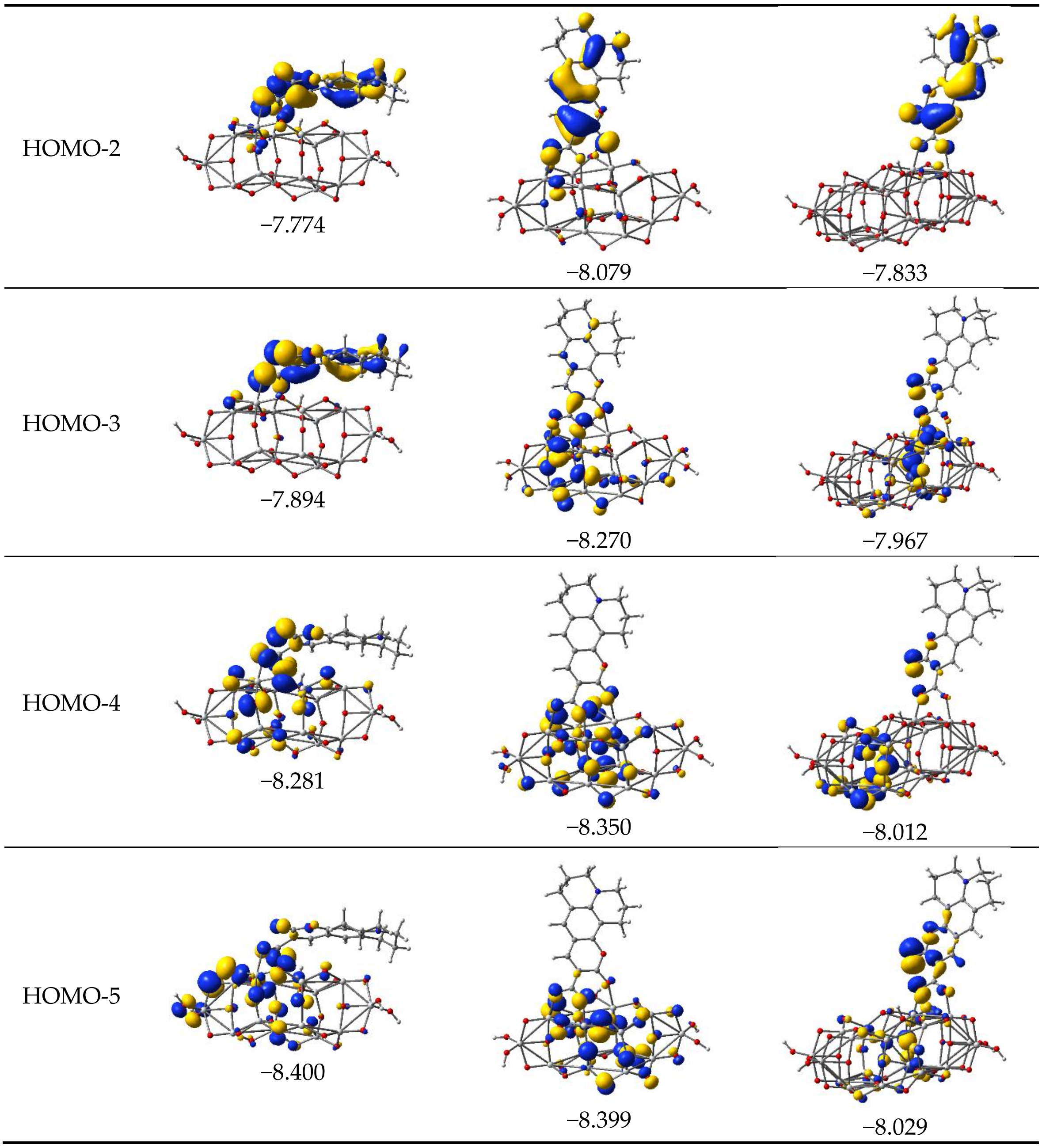

Figure S3. Isodensity surfaces (0.03 e/bohr^3) of the key molecular orbitals of C343 dye molecule adsorbed on the $Ti_{14}O_{30}H_4$ cluster via two or three O-Ti bonds, or on the $Ti_{24}O_{50}H_4$ cluster via the bidentate bridge binding mode, calculated by DFT at B3LYP/LANL2DZ level in water solvent.

Table S1. Energy, wavelength, oscillator strength and composition of the most intense optical transition for C343 dye molecule adsorbed on the $Ti_nO_{2n+2}H_4$ clusters (n = 14, 24), calculated by TDDFT at the B3LYP/LANL2DZ level in water solvent.

Dye	*E* (eV)	λ (nm)	*f*	Composition
C343-$Ti_{14}O_{30}H_4$, 2-bonds	1.97	630.4	0.029	HOMO→LUMO (26%), HOMO→LUMO+1 (71%)
	2.15	575.4	0.015	HOMO→LUMO+2 (88%)
	2.44	507.5	0.181	HOMO→LUMO+5 (61%), HOMO→LUMO+7 (23%)
	3.02	410.6	0.286	HOMO→LUMO+15 (32%), HOMO→LUMO+16 (24%), HOMO→LUMO+17 (29%)
C343-$Ti_{14}O_{30}H_4$, 3-bonds	2.19	565.7	0.039	HOMO→LUMO (94%)
	2.41	514.1	0.218	HOMO→LUMO+3 (85%)
	2.52	491.9	0.138	HOMO→LUMO+4 (76%)
	3.04	408.1	0.133	HOMO→LUMO+13 (81%)
	3.36	369.4	0.144	HO-1→LUMO (68%)
C343-$Ti_{24}O_{50}H_4$,	2.18	568.8	0.190	HOMO→LUMO+2 (98%)
	2.45	505.1	0.171	HOMO→LUMO+6 (89%)
	2.67	464.5	0.111	HOMO→LUMO+13 (41%), HOMO→LUMO+14 (29%)
	3.01	411.4	0.146	HO-1→LUMO (15%), HOMO→LUMO+23 (24%), HOMO→LUMO+24 (50%)
	3.04	408.0	0.118	HOMO→LUMO+25 (51%), HOMO→LUMO+26 (26%)

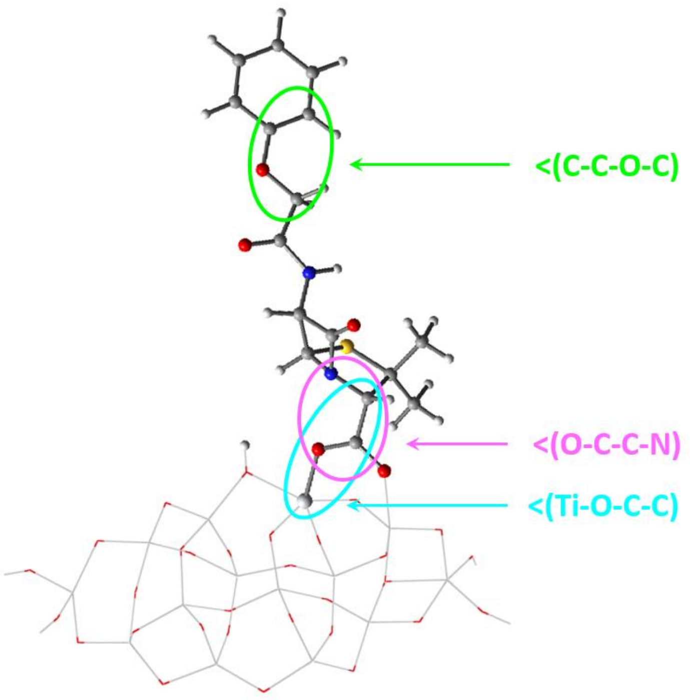

Figure S4. Structure of PV-$Ti_{14}O_{30}H_4$, to illustrate the geometrical parameters mentioned in Table 7: torsion angles of the PV relative to the Ti–O bond (cyan), to the carboxyl group (magenta), and of the rest of PV with respect to the phenyl group (green).

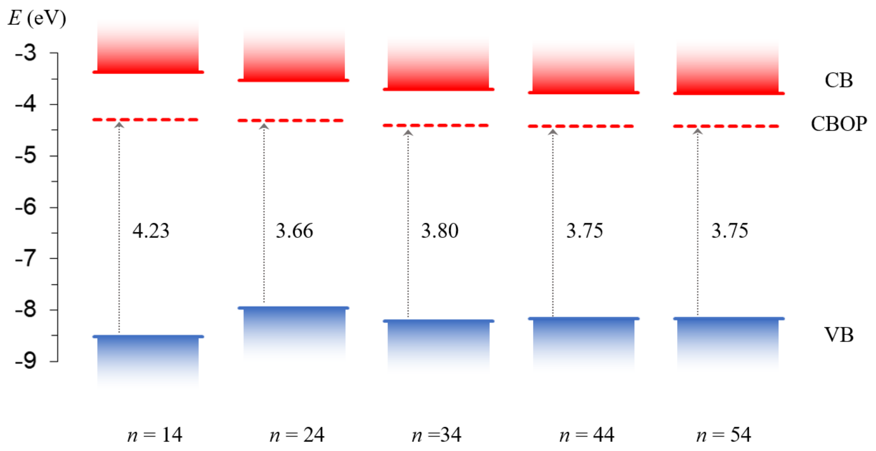

Figure S5. Valence band (VB) edge, conduction band oxidation potential (CBOP), and conduction band (CB) of the $Ti_nO_{2n+2}H_4$ clusters (*n* = 14, 24, 34, 44, 54), calculated at the DFT/B3LYP/LANL2DZ level in water solvent. HOMO-LUMO electronic transitions calculated by TD-DFT at the same level of theory (in eV).
